# Calcium‐Ion Insertion Chemistry in Tunneled α‐MnO_2_ Cathodes for Calcium Metal Batteries

**DOI:** 10.1002/advs.202508050

**Published:** 2025-09-12

**Authors:** Shuangshuang Cui, Yang Wang, Dechen Zeng, Zhaolin Lv, Andi Wang, Aobing Du, Zhenyou Li, Guanglei Cui

**Affiliations:** ^1^ Qingdao New Energy Shandong Laboratory Qingdao Institute of Bioenergy and Bioprocess Technology Chinese Academy of Sciences No. 189 Songling Road, Laoshan District Qingdao Shandong 266101 China; ^2^ Analytical and Testing Center South China University of Technology No. 381 Wushan Road, Tianhe District Guangzhou Guangdong 510640 China

**Keywords:** amorphization, calcium battery, diffusion kinetics, insertion mechanism, MnO_2_ cathode

## Abstract

Rechargeable calcium batteries (RCBs) are a promising sustainable energy storage technology with high theoretical energy density. However, their development is hindered by the lack of suitable cathodes that enable facile and reversible Ca^2+^ storage. This study investigates the Ca^2+^ storage mechanism in tunneled α‐K_0.03_MnO_2_ cathodes, revealing distinct electrochemical behaviors in two cell configurations using either activated carbon (AC) anodes or Ca metal anodes. While Ca^2+^ insertion/extraction dominates the charge storage process in Ca metal anode systems, negligible Ca^2+^ insertion occurs with AC anodes. Despite that, detailed mechanistic investigations of the Ca metal anode systems indicate sluggish Ca^2+^ diffusion within the α‐K_0.03_MnO_2_ framework, leading to irreversible cation trapping. Moreover, progressive Ca^2+^ accumulation causes deep calciation that triggers irreversible phase transition into CaMn_2_O_4_ and eventually complete structural degradation. To address these issues, a composite cathode combining ultrasmall, low‐crystallinity MnO_2_ nanoparticles with graphene oxide (u‐MnO_2_@GO) is developed, demonstrating improved Ca^2+^ diffusion kinetics, enhanced cycling stability over 60 cycles, and superior rate capability up to 50 mA g^−1^. This work provides critical insights into Ca^2+^ storage mechanisms in oxide cathodes and offers effective strategies for designing high‐performance cathodes for RCBs.

## Graphical abstract

α‐MnO_2_ cathodes demonstrate distinct electrochemical behavior and Ca^2+^ storage capabilities depending on whether they are paired with activated carbon anodes or Ca metal anodes. Ca^2+^ insertion/extraction is only feasible when α‐MnO_2_ cathodes are cycled against Ca metal anodes. Nevertheless, the sluggish Ca^2+^ diffusion leads to irreversible Ca^2+^ trapping, which can be mitigated through the development of an ultrasmall low‐crystalline MnO_2_ structure.

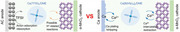

## 1 Introduction

The growing demand for electrochemical energy‐storage devices with higher energy density and reduced environmental impact has motivated the exploration of battery chemistries beyond the current lithium‐ion batteries (LIBs).^[^ ^1^ ^]^ Among these alternatives, rechargeable calcium batteries (RCBs) have recently gained significant research interest.^[^ ^2^, ^3^ ^]^ From a resource perspective, calcium (Ca) is the fifth most abundant element in the Earth's crust, ensuring a low‐cost and sustainable supply for large‐scale energy storage applications. Electrochemically, RCBs utilize a Ca metal anode, which theoretically offers high volumetric and gravimetric capacities of 2072 mAh cm^−3^ and 1337 mAh g^−1^, respectively, along with a low reduction potential of −2.87 V versus the standard hydrogen electrode (SHE).^[^ ^4^ ^]^ Notably, the reduction potential of Ca is only 0.17 V higher than that of Li, allowing RCBs to provide a cell voltage comparable to that of LIBs and exceeding that of other multivalent batteries with the same cathode applied.

Despite their advantages, RCBs remain in the early stage of development. One of the bottleneck issues is the lack of suitable cathode materials that facilitate reversible Ca^2+^ storage. The charge density of Ca^2+^ is similar to that of Li^+^ (∼52 C mm^−3^), resulting in a weaker Coulombic interaction with the host lattice, which theoretically allows for faster diffusion kinetics and higher reversibility compared to other charge‐dense multivalent carriers (e.g., Mg^2+^, Zn^2+^, Al^3+^). However, experimental results show that even typical layered hosts, such as TiS_2_, with a “softer” crystal lattice, suffer from sluggish kinetics during Ca^2+^ intercalation.^[^ ^5^ ^]^ Possible explanations for this include the large ionic radius of Ca^2+^ (1.14 Å) that induces steric hindrance and strong repulsion among adjacent charge carriers. In addition, the bivalent nature of Ca^2+^ complicates local charge compensation within the host structures.^[^ ^6^ ^]^

In the quest for viable cathode materials, research has primarily diverged into two key directions. Given the similar ionic radii of Ca^2+^ and Na^+^ (1.14 Å vs 1.16 Å), significant efforts have been directed toward exploring Ca^2+^ storage in well‐established Na^+^‐insertion compounds, such as Prussian blue analogues (PBAs)^[^ ^7^, ^8^ ^]^ and NASICON‐type polyanionic compounds.^[^ ^9^, ^10^, ^11^, ^12^ ^]^ Alternatively, transition metal chalcogenides, including oxides (Mg_0.25_V_2_O_5_·H_2_O, Ca_x_MoO_3_, (NH_4_)_2_V_7_O_16_)^[^ ^13^, ^14^, ^15^ ^]^ and sulfides (CuS, VS_4_),^[^ ^16^, ^17^ ^]^ have been investigated. Despite these efforts, most of the studied materials still exhibit unsatisfactory Ca^2+^ storage performance, particularly in terms of both cycling stability and rate performance.

Recently, MnO_2_ compounds have received considerable attention, mainly owing to their high redox potentials, high theoretical capacity, ability to host multivalent charge carriers, and the cost‐effectiveness of Mn, being a promising candidate for Ca^2+^ insertion host.^[^ ^18^, ^19^ ^]^ Compared with other high‐voltage cathodes such as Prussian blue analogues and polyanionic compounds, MnO_2_ does not contain other competitive charge carrier (e.g., Na^+^), which is more mobile than Ca^2+^, profiting the experimental analysis. MnO_2_ features various polymorphs, which are formed by corner‐ or edge‐sharing manganese octahedral [MnO_6_] units. The arrangement of these [MnO_6_] units gives rise to either layered or tunneled structures. An et al. investigated Ca^2+^ storage performance across different MnO_2_ polymorphs and found that δ‐MnO_2_ delivers the highest initial capacity.^[^ ^20^ ^]^ However, the intercalation of large‐sized Ca^2+^ leads to structural amorphization accompanied by Mn^2+^ dissolution, leading to active material loss and changes in the local coordination environment. This structural degradation is responsible for the capacity degradation of MnO_2_ cathode. These challenges can be partially mitigated through the co‐intercalation of Ca^2+^ and solvent into a layered Mg_0.15_MnO_2_ cathode, which, however, leads to electrolyte consumption.^[^ ^21^ ^]^ In contrast to the layered structure, tunneled α‐MnO_2_ exhibits enhanced cycling stability, albeit with a lower capacity.^[^ ^20^ ^]^ Despite these advancements, critical questions remain as to whether the tunneled structure is more tolerable against Ca^2+^ insertion, and what is the charge storage mechanism of α‐MnO_2_ in the Ca‐based batteries.

More critically, the majority of studies on cathode materials have been conducted in model cell configurations that utilize capacitive activated carbon (AC) anodes and electrolytes incompatible with Ca metal anodes. It is important to note that the desolvation of Ca^2+^ at the cathode‐electrolyte interfaces typically encounters a high energy barrier, which is largely dictated by the solvation structures within different electrolytes.^[^ ^22^ ^]^ In such cell configurations, other more mobile species, such as H⁺, may be generated due to electrolyte decomposition.^[^ ^23^ ^]^ These species can act as the primary charge carriers, dominating the subsequent insertion process and leading to undesired reaction pathways.^[^ ^24^ ^]^ Consequently, it remains unclear whether the charge storage mechanisms and Ca^2+^ storage capabilities observed in the model systems can be directly translated to the cell configurations employing Ca metal anodes or to practical RCBs.

This study aims to address the abovementioned concerns by investigating the Ca^2+^ insertion process in tunneled α‐MnO_2_. First, the electrochemical performance of the α‐MnO_2_ cathode and its compositional changes during charge–discharge cycles were compared in two distinct cell configurations utilizing either AC anodes or Ca metal anodes. The results reveal significant discrepancies between the electrochemical data and Ca content within the host structure when AC anodes are used. In contrast, when Ca metal anodes are employed, the Ca^2+^ insertion/extraction reaction dominates the charge storage process in the α‐MnO_2_ cathodes. However, irreversible Ca trapping was observed, leading to severe capacity fading. Mechanistic investigations indicate that the sluggish diffusion of Ca^2+^ within the tunneled structure hinders the following de‐/calciation process. Therefore, an ultrasmall MnO_2_ nanomaterial (u‐MnO_2_) with a short‐range ordering structure was designed to enhance the solid diffusion kinetics of Ca^2+^ while reducing the diffusion lengths. To achieve improved electronic conductivity for u‐MnO_2_, a u‐MnO_2_@graphene oxide (u‐MnO_2_@GO) composite cathode was developed. The u‐MnO_2_@GO cathode enables reversible Ca^2+^ de‐/insertion, achieving a capacity of ≈110 mAh g^−1^ and demonstrating stable cycling over 60 cycles in RCBs.

## 2 Results and Discussion

### 2.1 Structural Characterization of the α‐MnO_2_

**Figure 1:**
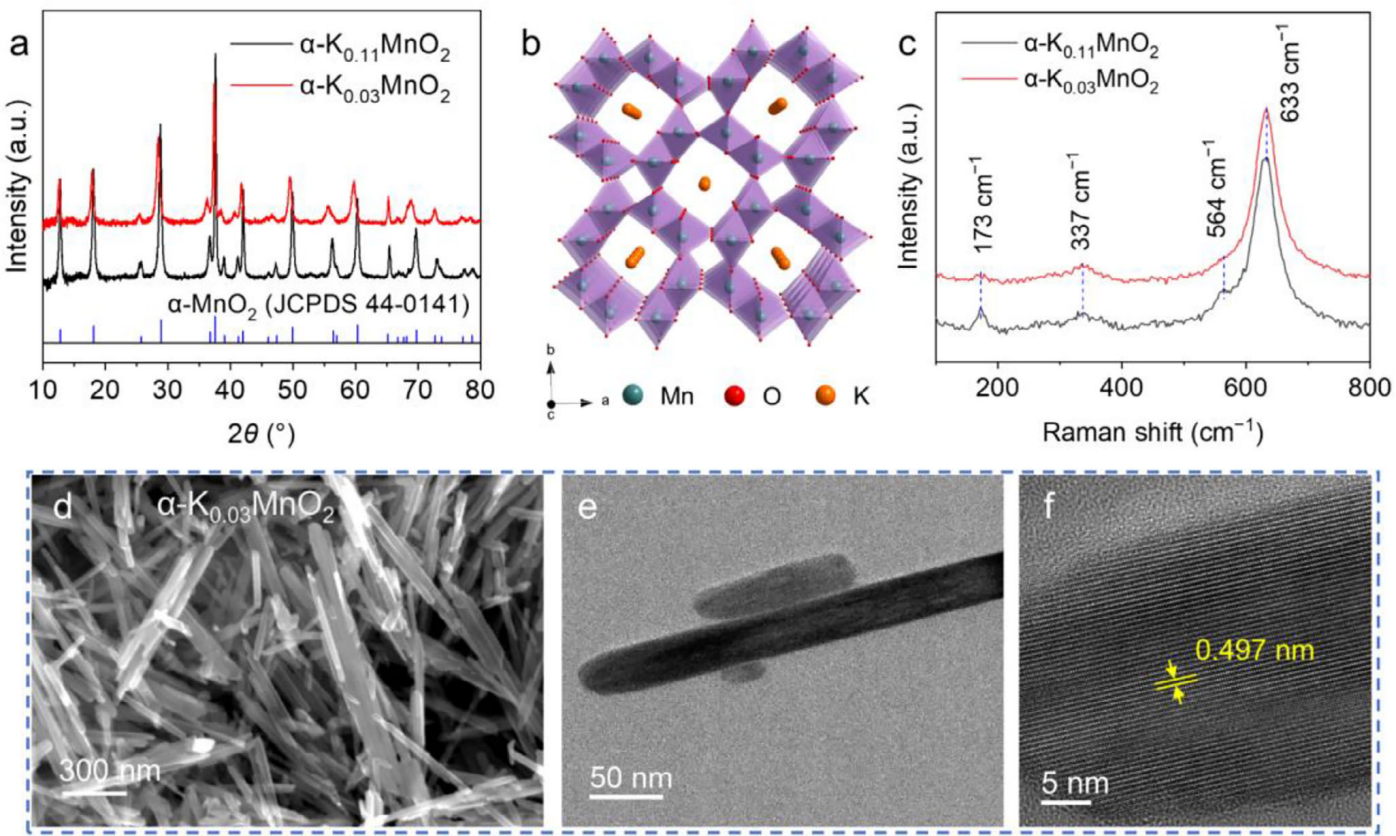
Structural and morphological characterization of the as‐synthesized and the K^+^‐deficient α‐MnO_2_ samples. a) XRD pattern. b) Crystallographic structure model. c) Raman spectra. d) SEM image. e) TEM image. f) HRTEM image.

The as‐prepared α‐MnO_2_ samples were characterized by X‐ray diffraction (XRD). As depicted in **Figure** **1**a, all diffraction peaks can be indexed to the tetragonal α‐MnO_2_ phase. The crystal structure of α‐MnO_2_ is illustrated in Figure 1b, which features 1D 2 × 2 and 1 × 1 tunnels formed by [MnO_6_] octahedras. The large 2 × 2 tunnels, with dimensions of 4.6 Å × 4.6 Å, are capable of accommodating suitable cations.^[^ ^25^ ^]^ Since KMnO_4_ was used as the precursor, a certain amount of K^+^ was incorporated into the α‐MnO_2_ structure. Inductively coupled plasma optical emission spectroscopy (ICP‐OES) analysis revealed that the as‐prepared α‐MnO_2_ contains 0.11 K^+^ per formula unit (denoted as α‐K_0.11_MnO_2_). To minimize the influence of K^+^ on the Ca^2+^ storage performance, the α‐K_0.11_MnO_2_ was subjected to acid treatment to remove K^+^. After the acid treatment, the K^+^ content was reduced to 0.03 per formula unit (denoted as α‐K_0.03_MnO_2_). XRD results confirmed that the α‐MnO_2_ phase was maintained after the extraction of most of K^+^, while a slight peak shift toward lower 2*θ* degrees was observed due to the tunnel contraction.^[^ ^26^ ^]^

The Raman spectra in Figure 1c show typical vibrational modes of α‐MnO_2_, with peaks centered at 173, 337, 564, and 633 cm^−1^. The peak at 173 cm^−1^ corresponds to the external vibration resulting from the translational motion of MnO_6_ octahedra, while the band at 337 cm^−1^ is associated with Mn─O bending vibrations. The strong peak at 633 cm^−1^ and the shoulder peak at 564 cm^−1^ are attributed to the symmetric stretching vibrations of Mn─O bonds in MnO_6_ octahedra.^[^ ^27^, ^28^ ^]^ Notably, the intensity of the peak at 564 cm^−1^ is sensitive to the type and amount of cations occupying the tunnels of α‐MnO_2_.^[^ ^29^ ^]^ Therefore, the significant reduction in the intensity of the 564 cm^−1^ peak in the spectrum of the K^+^‐deficient α‐K_0.03_MnO_2_ compared to that of α‐K_0.11_MnO_2_, can be ascribed to the removal of K^+^.

Scanning electron microscopy (SEM) and transmission electron microscopy (TEM) images (Figure 1d,e) reveal that the α‐K_0.03_MnO_2_ samples exhibit a nanorod morphology, with diameters ranging from 20–30 nm and lengths extending up to several micrometres. High‐resolution TEM (HRTEM) image (Figure 1f) further demonstrates that α‐K_0.03_MnO_2_ possesses a long‐range ordered lattice structure along the (001) direction. The measured lattice spacing of 0.497 nm corresponds to the (200) plane. Notably, the morphology and lattice structure of α‐K_0.03_MnO_2_ are consistent with those of the K^+^‐containing α‐K_0.11_MnO_2_ samples (Figure S1, Supporting Information). In particular, the identical ordered lattice fringe of the (200) plane along the length direction confirms that the crystal structure and crystallinity of α‐MnO_2_ were maintained after the extraction of K^+^. The selected area electron diffraction (SAED) pattern (Figure S2, Supporting Information) indicates that both the α‐K_0.03_MnO_2_ and α‐K_0.11_MnO_2_ nanorods are single crystalline with higher crystallinity.

### 2.2 Different Charge Storage Mechanisms in the α‐MnO_2_ Cathode

**Figure 2:**
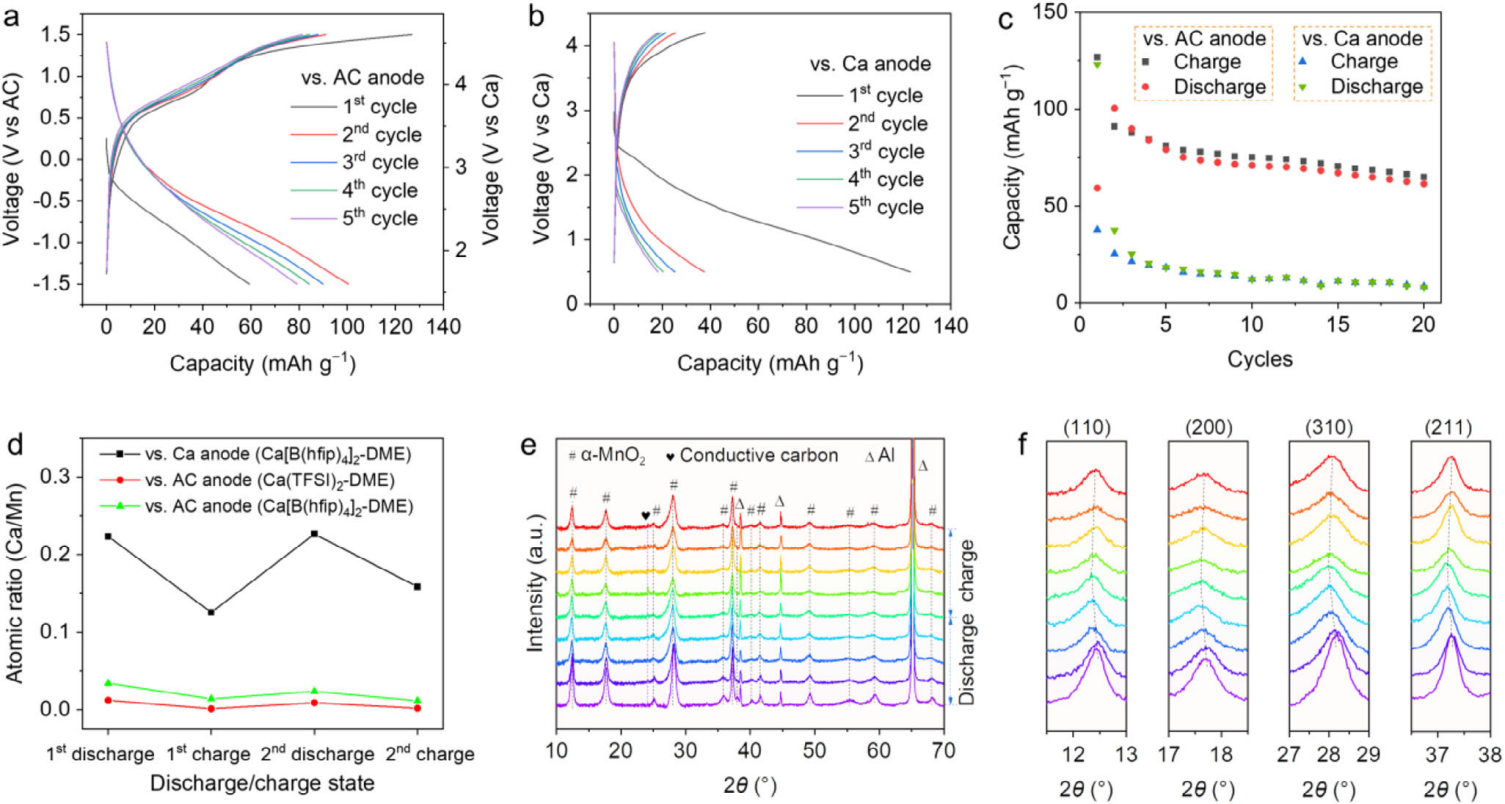
Electrochemical measurements, elemental analysis, and structural characterization of the α‐K_0.03_MnO_2_ cathodes in the two cell configurations. Galvanostatic charge–discharge (GCD) curve of α‐K_0.03_MnO_2_ cathode at a current density of 50 mA g^−1^ in the cells with an AC anode (a), or with a Ca anode (b), and the corresponding cycling performance (c). d) EDS results during the first two discharge/charge cycles. e) Ex situ XRD patterns of the α‐K_0.03_MnO_2_ cathode during the first discharge/charge cycle in the Ca cells (at a current density of 10 mA g^−1^). f) (110), (200), (310), and (211) peaks of α‐MnO_2_ in selected 2*θ* ranges.

To evaluate the Ca^2+^ storage performance of the α‐K_0.03_MnO_2_ cathode, two cell configurations were compared: one utilizing an AC anode and the other employing a Ca metal anode. In the AC‐based cells, the α‐K_0.03_MnO_2_ cathode was cycled between −1.5 and 1.5 V (vs AC) in a 0.5 m Ca(TFSI)_2_/DME electrolyte. Using ferrocene as an internal reference for calibration (Figure S3, Supporting Information), this voltage window corresponds to 1.6 − 4.6 V versus Ca. As shown in **Figure** **2**a, the α‐K_0.03_MnO_2_ cathode delivered an initial discharge capacity of 59.3 mAh g^−1^ at a current density of 50 mA g^−1^. The discharge capacity increased to 100.5 mAh g^−1^ by the second cycle but gradually decreased to 79.1 mAh g^−1^ after five cycles. In contrast, when paired with a Ca metal anode, the α‐K_0.03_MnO_2_ cathode exhibited a higher initial discharge capacity of 123.1 mAh g^−1^, however, this was accompanied by a rather low charge capacity of 37.8 mAh g^−1^ (Figure 2b). The discharge capacity dropped significantly to 37.5 mAh g^−1^ by the second cycle and continued to decline with subsequent cycles. As illustrated in Figure 2c, the α‐K_0.03_MnO_2_ cathode exhibits superior capacity and cycling stability with the AC anode compared to the Ca metal anode. Notably, this performance difference is not attributable to the different voltage window applied. As shown in Figure S4 (Supporting Information), cycling the K_0.03_MnO_2_ cathode in the voltage range of −2.0 to 1.2 V versus AC (corresponding to 1.1 to 4.3 V vs Ca) yielded similar performance (cf. Figure 2a). However, the cell sometimes shows over‐discharging, likely due to the potential drift of the AC anode. As a result, the voltage range of −1.5 to 1.5 V (vs AC) was chosen for the following studies.

The redox activity of the α‐K_0.03_MnO_2_ cathode in the two cell configurations was further investigated using cyclic voltammetry (CV) at a scan rate of 0.2 mV s^−1^. As shown in Figure S5a (Supporting Information), the α‐K_0.03_MnO_2_ cathode in the cells with an AC anode exhibited a broad reduction peak at −1.05 V and a strong oxidation peat at 0.93 V. In comparison, for the α‐K_0.03_MnO_2_ cathode versus a Ca anode (Figure S5b, Supporting Information), a two‐step reduction process was observed during the first scan cycle, with peaks centered at 2.38 and 0.79 V. This behavior can be attributed to the intercalation of multiple Ca^2+^.^[^ ^30^ ^]^ The corresponding anodic scan revealed only a broad oxidation peak ≈3.01 V with a lower response current, suggesting incomplete extraction of Ca^2+^ from the α‐K_0.03_MnO_2_ structure. The significant decrease in the current of the redox peaks during the second scan cycle indicates reduced redox activity, which aligns well with the capacity decay observed in Figure 2c.

To elucidate the stark contrast in the electrochemical properties and redox activity of the α‐K_0.03_MnO_2_ cathode in the two cell configurations, elemental analysis was conducted to identify the active charge carriers. Figure 2d presents the energy‐dispersive spectroscopy (EDS) measurements for the α‐K_0.03_MnO_2_ cathode during discharge/charge cycles. In the Ca cell, the Ca content was measured at 0.22 and 0.12 per formula unit in the first discharge and charge states, respectively. Despite the evident Ca trapping, reversible Ca intercalation and extraction were still observed during the second cycle. In contrast, elemental analysis of the α‐K_0.03_MnO_2_ cathode in the AC cells revealed negligible Ca content, unambiguously ruling out Ca^2+^‐related redox reactions.

To further exclude the influence of solvation structures in different electrolytes on the Ca^2+^ storage capability of the α‐K_0.03_MnO_2_ cathode, the 0.25 m Ca[B(hfip)_4_]_2_/DME electrolyte, used in the Ca cells, was also employed in the AC cells. As shown in Figure S6 (Supporting Information), the α‐K_0.03_MnO_2_ cathode in the AC cells with the Ca[B(hfip)_4_]_2_/DME electrolyte exhibited higher polarization and lower capacity compared to those using the Ca(TFSI)_2_‐based electrolyte. Nevertheless, the consistently low Ca content observed during cycling in the two electrolyte systems (Figure 2d) confirms the inability of the α‐K_0.03_MnO_2_ cathode to store Ca^2+^ in the AC cells.

In addition to chemical composition, structural changes in the α‐K_0.03_MnO_2_ cathode were also compared between the two cell configurations. Figure 2e displays the ex situ XRD patterns of the α‐K_0.03_MnO_2_ cathode during the first cycle in the Ca cells. The diffraction peaks of α‐K_0.03_MnO_2_ weakened and broadened during the discharge process, indicating reduced crystallinity due to Ca^2+^ intercalation. These peak changes persisted during the subsequent charge process, suggesting irreversible disordering of the α‐K_0.03_MnO_2_ structure. As shown in Figure 2f, the peaks at 12.44°, 17.69°, 28.18°, and 37.26°, corresponding to the (110), (200), (310), and (211) planes of α‐MnO_2_, gradually shifted to lower 2*θ* angles during discharge. This implies that Ca^2+^ storage in the α‐K_0.03_MnO_2_ cathode proceeded via a solid‐solution reaction, with tunnel expansion in the host structure caused by Ca^2+^ intercalation. Upon full charging, the peaks shifted back toward higher 2*θ* angles but did not fully return to their original positions, consistent with incomplete Ca^2+^ extraction. Notably, despite the absence of reversible Ca^2+^ (de)intercalation in the AC cells, the XRD patterns of the α‐K_0.03_MnO_2_ cathode (Figure S7, Supporting Information) exhibited similar peak shifts and weakening during discharge/charge cycles.

The results presented above demonstrate that the electrochemical behavior and performance of the α‐K_0.03_MnO_2_ cathode are strongly influenced by the choice of anode and electrolyte. While the evolution of characteristic diffraction peaks reveals insertion reactions in both cell configurations, the substantial variation in Ca content observed in the Ca cells provides compelling evidence for the Ca^2+^ storage in the α‐K_0.03_MnO_2_ cathode.

**Figure 3:**
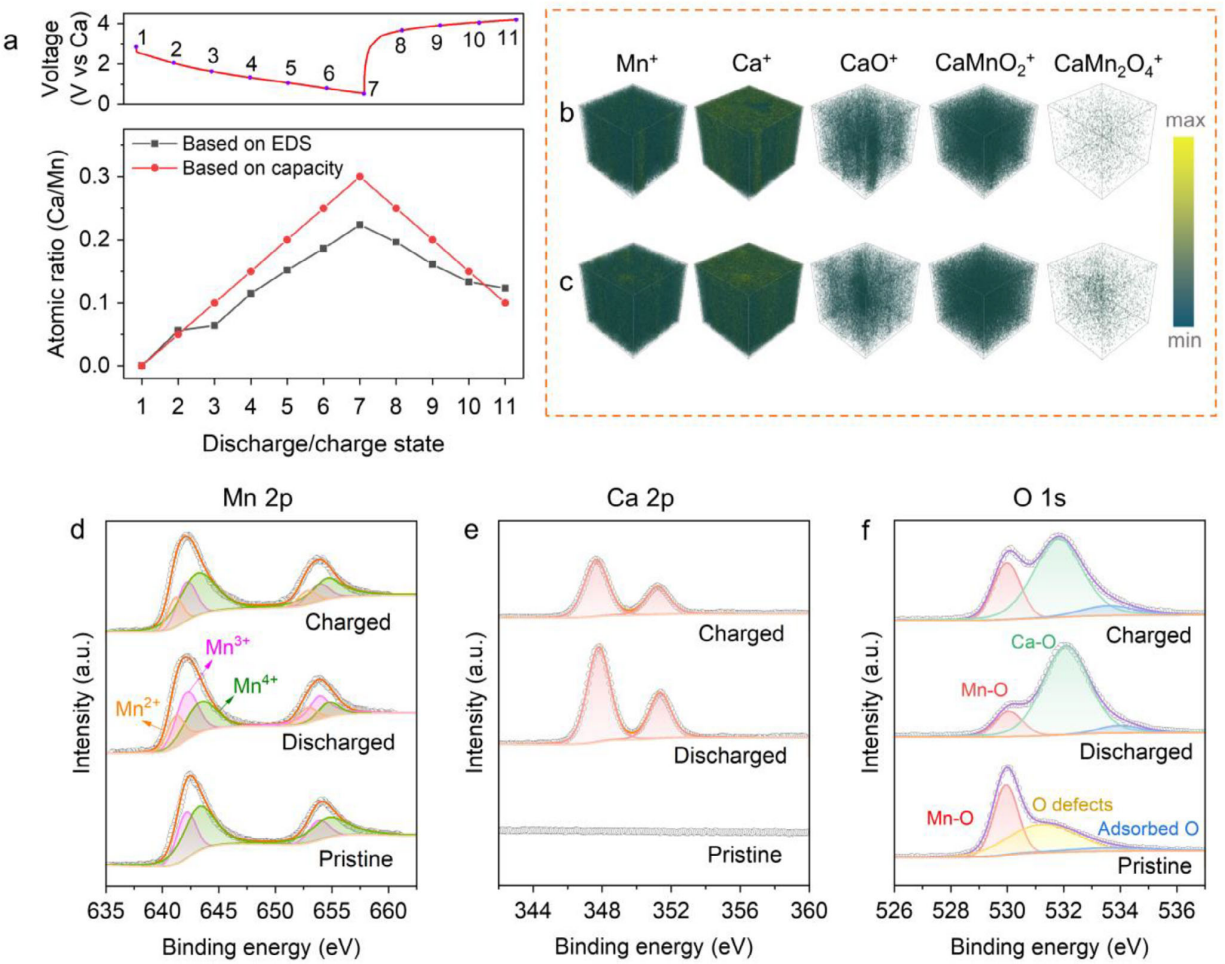
EDS, ToF‐SIMS, and XPS characterization of the α‐K_0.03_MnO_2_ cathode during the first discharge/charge cycle in the Ca cells (at a current density of 10 mA g^−1^). a) EDS results and the corresponding GCD curve. 3D distributions of representative secondary ion species obtained by sputtering the discharged cathode (b) and the charged cathode (c). XPS results of Mn 2p spectra (d), Ca 2p spectra (e), O 1s spectra (f). The peak at 531.47 eV in (f) for pristine α‐K_0.03_MnO_2_ cathode is aligned to the oxygen defects.^[^ ^32^ ^]^

To further investigate the underlying mechanism, additional elemental analysis was conducted. Specifically, α‐K_0.03_MnO_2_ cathodes at intermediate states were collected after every 0.1 e^−^ transfer during the discharge–charge process. EDS results of the ex situ cathodes (**Figure** **3**a) reveal that the Ca/Mn ratio increases almost linearly from 0 to 0.22 during discharge. The Ca content measured by EDS aligns reasonably well with the values calculated based on capacity, confirming that Ca^2+^ intercalation dominates the electrochemical process of the α‐K_0.03_MnO_2_ cathode. Upon charging, the Ca/Mn ratio gradually decreases as Ca^2+^ is extracted. However, ≈0.12 Ca^2+^ remains irreversibly trapped within the structure after fully charged, highlighting the partial irreversibility of the Ca^2+^ de‐/intercalation process. Note that the K/Mn ratio almost remains unchanged throughout electrochemical cycling (Figure S8, Supporting Information), indicating that the residual K^+^ in α‐K_0.03_MnO_2_ cathode functions as structural pillars rather than participating in charge transfer processes. Further evidence for Ca^2+^ insertion was obtained through time‐of‐flight secondary‐ion mass spectrometry (ToF‐SIMS). ToF‐SIMS depth profiling of the discharged sample (Figure 3b; Figure S9a, Supporting Information) showed 3D distributions of Mn^+^, Ca^+^, CaO^+^, CaMnO_2_^+,^ and CaMn_2_O_4_^+^ secondary ions, corroborating the formation of Ca_x_MnO_2_ and CaMn_2_O_4_. Notably, the detection of postspinel CaMn_2_O_4_ suggests a partial phase transition, which will be discussed in detail in the TEM section. In agreement with EDS results, residual Ca^+^, CaO^+^, CaMnO_2_^+,^ and CaMn_2_O_4_^+^ secondary ions were still observed in the charged sample (Figure 3c; Figure S9b, Supporting Information), further confirming irreversible Ca^2+^ entrapment.

XPS measurements were performed to investigate the valence state and composition of the α‐K_0.03_MnO_2_ cathode during the discharge/charge process. In the pristine α‐K_0.03_MnO_2_ (Figure 3d), the Mn 2p spectrum displays characteristic doublets of Mn^3+^ at 642.15/653.84 eV and Mn^4+^ at 643.57/654.69 eV. After discharge, the intensity of the Mn^4+^ peaks decreases, while the Mn^3+^ signals intensify, indicating Mn reduction. Additionally, new peaks corresponding to Mn^2+^ emerge at 641.17 and 652.84 eV, confirming further reduction due to Ca^2+^ intercalation. This is corroborated by the appearance of Ca 2p peaks at 347.83 and 351.40 eV in the Ca 2p spectra (Figure 3e). Upon recharge, Mn undergoes re‐oxidation, evidenced by the weakening of Mn^3+^ peaks and the enhancement of Mn^4+^ peaks. The O1s spectra (Figure 3f) provide additional insights: a new peak at 532.08 eV emerges upon discharge, which is attributed to the Ca─O bond arising from Ca_x_MnO_2_ and CaMn_2_O_4_. This is accompanied by a decrease in the Mn─O peak intensity at 530.06 eV. Even after charging, the Ca─O signal persists with appreciable intensity, though the Mn─O/Ca─O ratio increases. Meanwhile, the Ca 2p peaks weaken but remain detectable, confirming irreversible Ca^2+^ entrapment within the α‐K_0.03_MnO_2_ or CaMn_2_O_4_ structure.

The local structure evolution of the α‐MnO_2_ cathode during Ca^2+^ intercalation/extraction was further investigated using Raman spectroscopy. As shown in Figure S10 (Supporting Information), the strong peak at 633 cm^−1^, associated with the Mn─O symmetric stretching vibrations of the MnO_6_ octahedral chains, exhibits a redshift during discharge. This shift is attributed to the expansion of the tunnels caused by Ca^2+^ insertion, which leads to the compression of the [MnO_6_] octahedras.^[^ ^31^ ^]^ Upon recharge, the extraction of Ca^2+^ induces a blueshift of the peak. Besides, the shoulder peak at 566 cm^−1^, which is sensitive to cation occupancy within the tunnels, gradually increases in intensity during discharge owing to Ca^2+^ intercalation. However, this enhanced peak does not fully recover after charging, further indicating the irreversible entrapment of Ca^2+^.

In addition to Ca^2+^ entrapment, a critical concern is whether oxygen redox occurs at high voltages, as the charging cutoff (4.2 V vs Ca) is approaching the oxidative stability limit of ethereal solvents, which could potentially lead to the formation of oxide or peroxide. To clarify this, we further analyzed the ToF‐SIMS data and observed a spatial correlation between the 3D distributions of Ca^+^, CaO^+,^ and CaMnO_2_^+^ (Figure 3b), confirming that the Ca‐based oxide fragments mainly origin from the Ca^2+^ intercalation into α‐K_0.03_MnO_2_ cathode rather than oxygen redox chemistry.^[^ ^33^ ^]^ This conclusion is further supported by XPS analysis: the evolution of Ca 2p peaks and the Ca─O peak in the O1s spectra correlate well with changes in Mn valence states and the corresponding peak intensity, demonstrating that Ca^2+^ interacts directly with Mn rather than Ca─O reactions. Moreover, Raman spectra in Figure S10 (Supporting Information) revealed no detectable signals corresponding to CaO (358 and 689 cm^−1^) or CaO_2_ (350 and 850 cm^−1^),^[^ ^34^, ^35^ ^]^ ruling out the formation of such species. The combined evidence from ToF‐SIMS, XPS, and Raman spectra conclusively excludes oxygen participation in the redox processes, confirming that the electrochemical behavior is governed mainly by Ca^2+^ intercalation and Mn redox.

Despite demonstrating high capacity and cycling stability, the α‐K_0.03_MnO_2_ cathode exhibits poor Ca^2+^ storage capability in the AC cells. Considering that the insertion of multivalent ions can be largely influenced by the solvation structure of shuttling ions in the electrolyte,^[^ ^36^ ^]^ an alkyl carbonate‐based electrolyte consisting of 0.8 m Ca(TFSI)_2_ in a mixture of ethylene carbonate (EC), propylene carbonate (PC), dimethyl carbonate (DMC), and ethyl methyl carbonate (EMC) in 3:3:2:2 volume ratio was also employed for α‐K_0.03_MnO_2_ cathode in the AC cells. As shown in Figure S11 (Supporting Information), although the cell delivers a stable capacity of ≈65 mAh g^−1^, EDS results show almost no Ca^2+^ uptake, which are same with the ether‐based electrolyte. A similar phenomenon has been reported for V_2_O_5_ cathodes in divalent Mg and Ca batteries with carbonate‐based electrolytes, where the electrochemical process is dominated by the formation of protonated phases rather than the intercalation of divalent ions (Mg^2+^, Ca^2+^).^[^ ^24^ ^]^ The proton source can be either solvent or residual H_2_O. To exclude the possibility of H^+^ producing from residual H_2_O, all electrolyte compounds and the solvents, as well as AC anodes were rigorously dried before the assemble of AC anode cells. For DME, which was used in both Ca‐based and AC‐based cells, Karl Fisher titration confirmed water content below 20 ppm. This result further supports the dehydrogenation of the organic solvents. In fact, the dehydrogenation of carbonate solvents was recently identified.^[^ ^37^ ^]^ Compared to carbonate, ethereal solvents typically exhibit a lower oxidation stability and thereby easier dehydrogenation can be expected. However, protonation reactions in ether‐based electrolytes have not been reported and require further exploration. Considering the difference in ion mobility between H^+^ and Ca^2+^, the migration kinetics of the active charge carriers in AC and Ca anode system were further examined using galvanostatic intermittent titration technique (GITT). As shown in Figure S12 (Supporting Information), the values for the ion diffusion coefficient in AC anode cell are significantly higher than those in Ca anode cell at low depth of discharge (DoD), which supports the hypothesis of H⁺ intercalation in AC anode cells. However, the ion diffusion coefficient gradually reduced to the same order of magnitude with that in Ca anode cell with the increase of DoD. This phenomenon may be related to the dehydrogenation process of solvents and needs further exploration. Thus, the critical question arises regarding the relationship between the domination of charge carriers (Ca^2+^ or H^+^) and the anode (Ca or AC) system for α‐MnO_2_ cathodes. One plausible explanation is that H^+^ may be consumed by forming compounds like CaH_2_ with Ca metal anodes due to the highly reductive nature of Ca, while it could remain preserved and electrochemically active in AC‐based cells. Since the active H^+^ and Ca^2+^ are competing for inserting into the cathode in AC anode cells, the insertion of Ca^2+^ can still be realized by designing the host lattice of cathode material or the solvation structure of Ca^2+^ through providing rapid diffusion kinetics for Ca^2+^. This is beyond the scope of the present study and warrants further investigation.

### 2.3 Degradation Mechanism of the α‐MnO_2_ Cathode Against Ca Metal Anode

**Figure 4:**
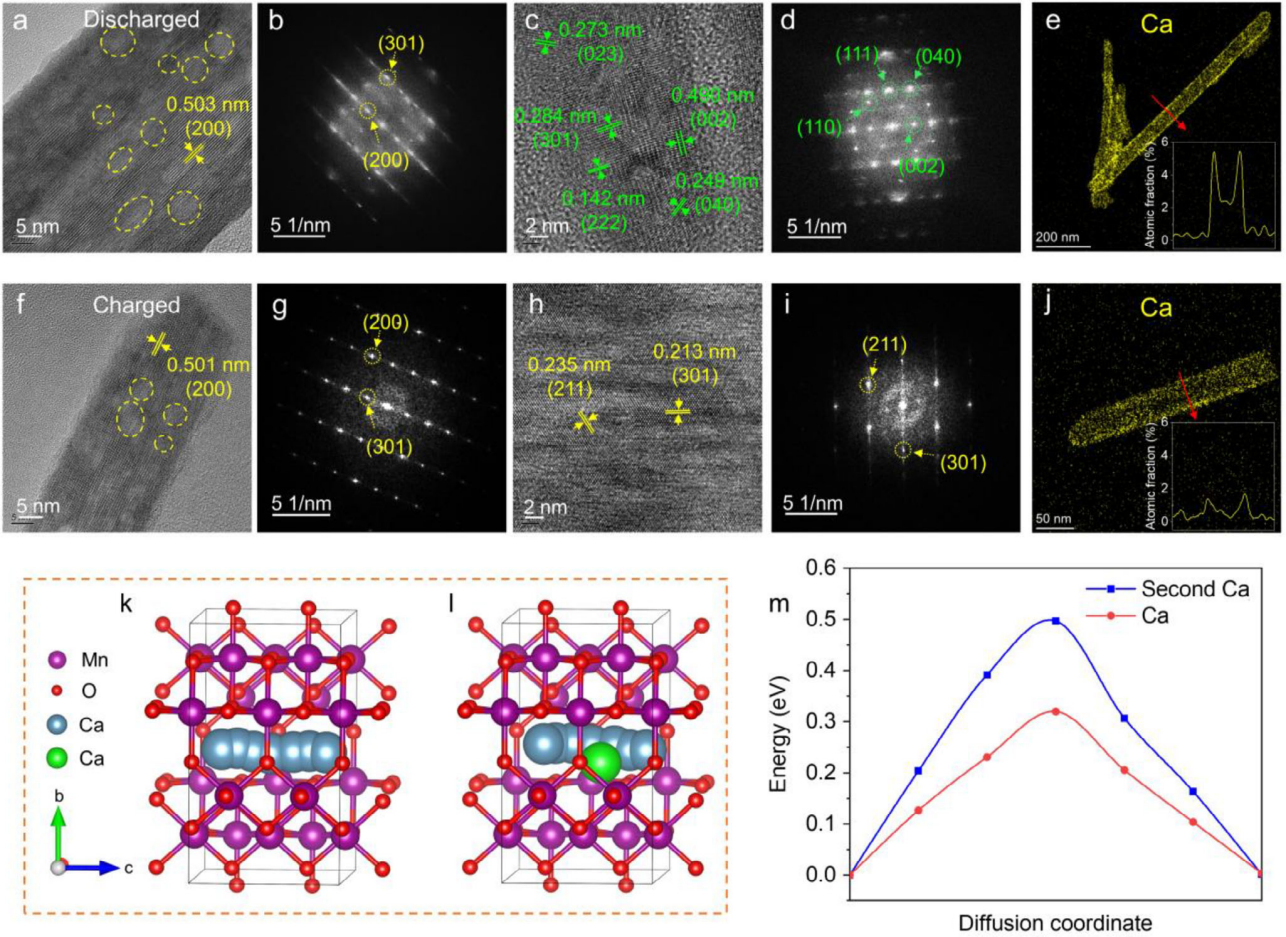
TEM characterization of α‐K_0.03_MnO_2_ cathode during the first discharge/charge cycle (at a current density of 10 mA g^−1^) and DFT calculation results. a,c) HRTEM images, b,d) the corresponding FFT patterns, and e) Ca distribution after the first discharge. f,h) HRTEM images, g,i) the corresponding FFT patterns, and j) Ca distribution after the first charge. The yellow and green marks of the lattice plane and lattice distance in (a‐i) correspond to α‐MnO_2_ and CaMn_2_O_4_, respectively. k,l) Energetically favorable diffusion pathways for the first and second Ca^2+^, respectively. m) The corresponding energy profiles.

While the α‐K_0.03_MnO_2_ cathode exhibits Ca^2+^ insertion with certain reversibility in the Ca cells, the rapid capacity fade and irreversible cation trapping motivate further investigation into the structural degradation. Given that XRD showed no evidence of phase transformations, we conducted detailed TEM characterization to elucidate the local structural and morphological changes in cycled α‐K_0.03_MnO_2_ particles. As shown in Figure S13a (Supporting Information), the nanorod morphology of α‐K_0.03_MnO_2_ well maintained after discharge. Typical HRTEM image in **Figure** **4**a revealed the persistence of the lattice structure of α‐K_0.03_MnO_2_ with a lattice spacing of 0.503 nm corresponding to its (200) planes. The corresponding FFT pattern (Figure 4b) displayed reflections from the (301) and (200) planes. Notably, the long‐range ordered lattice structure of α‐K_0.03_MnO_2_ was interrupted by some disordered lattices (highlighted by the yellow rings in Figure 4a), indicative of amorphization resulting from Ca^2+^ insertion. This observation aligns with the broadened diffraction peaks of α‐MnO_2_ observed in XRD results. Furthermore, the majority of the nanorods exhibited a complete degradation of the α‐K_0.03_MnO_2_ lattice structure, fragmenting into several nanocrystallines (Figure 4c). The FFT pattern (Figure 4d) identified these nanocrystallines as postspinel CaMn_2_O_4_, consistent with the CaMn_2_O_4_^+^ secondary ions detected by ToF‐SIMS and the appearance of Ca─O peak in the XPS O 1s spectra. The absence of the CaMn_2_O_4_ phase in the XRD results can be attributed to its relatively short‐range ordered lattice structure. Furthermore, elemental mapping and EDS analysis of discharged α‐K_0.03_MnO_2_ (Figure 4e; Figure S13b, Supporting Information) reveal that Ca is predominantly aggregated in the surface region, resulting in a calcium‐rich shell relative to the bulk core. This heterogeneous distribution and “core–shell” structure stem from sluggish Ca^2+^ diffusion kinetics that restrict deep intercalation into the core region of the α‐K_0.03_MnO_2_ nanorods. A similar surface‐confined intercalation phenomenon has been reported for the calciation of NaV_2_(PO_4_)_3_ cathode.^[^ ^11^ ^]^ The beneficiation of Ca^2+^ in the shell of α‐K_0.03_MnO_2_ nanorods leads to the surface structure degradation, while the bulk structure maintains, consistent with the XRD results. Notably, the nanocrystallines and the characteristic lattice of CaMn_2_O_4_ were not detected for the discharged α‐K_0.03_MnO_2_ cathode when against AC anodes (Figure S14, Supporting Information), further confirming the Ca^2+^ storage inability of α‐K_0.03_MnO_2_ cathode in the AC cells.

**Figure 5:**
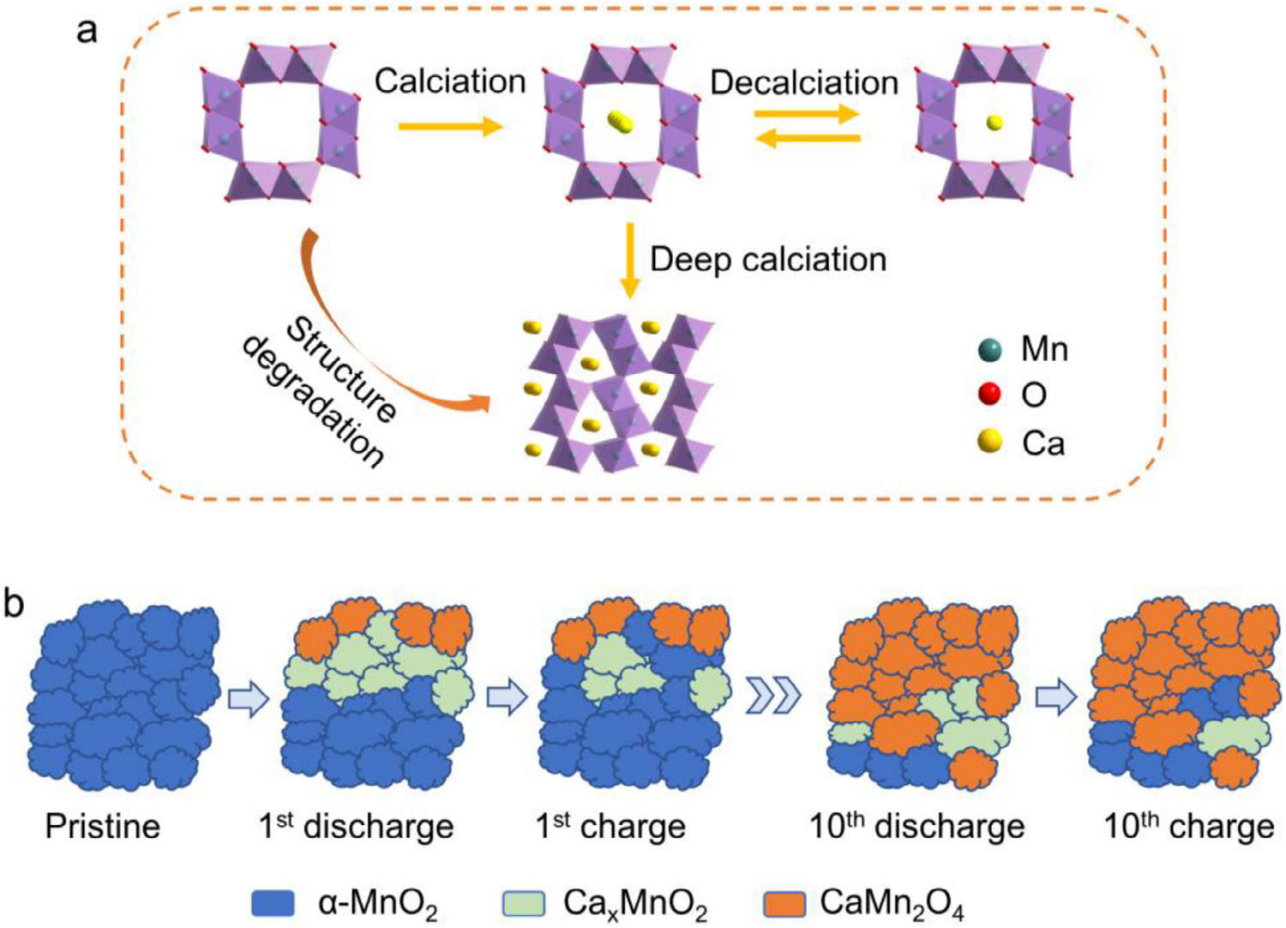
Reaction mechanism of α‐K_0.03_MnO_2_ cathodes in Ca cells. a) Ca^2+^ intercalation process in α‐K_0.03_MnO_2_ cathodes; b) structural evolution of α‐K_0.03_MnO_2_ cathodes during cycling.

According to previous studies, the structural evolution of α‐MnO_2_ is strongly influenced by the size and concentration of the charge carriers in the host structure. When Li^+^ are intercalated, the tunnelled structure of α‐MnO_2_ remains largely preserved, with Li^+^ preferentially storing at the off‐center positions of the Wyckoff 8h sites.^[^ ^38^ ^]^ In contrast, the larger Na^+^ tend to occupy near‐centered positions within the 2 × 2 tunnels, causing significant distortion of the host lattice as Na^+^ intercalation proceeds.^[^ ^39^, ^40^ ^]^ Lu et al. demonstrated that the tunnelled structure of α‐MnO_2_ becomes unstable upon Na^+^ insertion, leading to the formation of an intermediate NaMn_2_O_4_ phase, which further transforms into Mn_2_O_3_ and Na_2_O upon fully sodiation.^[^ ^39^ ^]^ A similar transformation mechanism was observed for Mg^2+^ insertion into α‐MnO_2_. Arthur et al. reported that α‐MnO_2_ cathodes first undergo a solid‐solution reaction upon Mg^2+^ insertion to form Mg_0.5_MnO_2_, which is thermodynamic unstable and eventually converts to an amorphous mixture of MgO and Mn_2_O_3_.^[^ ^41^ ^]^ To identify the preferred insertion sites for Ca^2+^ in α‐MnO_2_, density functional theory (DFT) calculations were performed. Similar to the behavior observed in Na‐ion batteries, Ca^2+^ prefers to occupy the 2a sites at the center of the 2 × 2 tunnels, as depicts in Figure S15 (Supporting Information). As illustrated in **Figure** **5**a, the continuous insertion of Ca^2+^ results in the degradation of the host structure and the formation of CaMn_2_O_4_. However, unlike the Na^+^ or Mg^2+^ case, further structural transformation into Mn_2_O_3_ and CaO was not observed.

After fully recharge, the nanorod morphology of α‐K_0.03_MnO_2_ remains well‐preserved (Figure S16a, Supporting Information). As illustrated in Figure 4f, the partially disordered lattice structure does not revert to its pristine state, consistent with the XRD results, indicating irreversible structural amorphization. Importantly, the transformation of α‐K_0.03_MnO_2_ into CaMn_2_O_4_ is nearly irreversible. Previous computational studies predict that decalciation of postspinel CaMn_2_O_4_ would require high voltages of 3.7 V and overcome substantial diffusion barriers above 1.8 eV, with experimental evidence confirming the impracticality of Ca^2+^ extraction.^[^ ^42^ ^]^ However, the characteristic lattice of CaMn_2_O_4_ nanocrystallines was not directly observed (Figure 4f–i), this absence likely results from the limit of the sample area detected by TEM. Despite that, EDS analysis (Figure 4j; Figure S16b, Supporting Information) confirms the presence of residual Ca^2+^ trapped within the α‐K_0.03_MnO_2_ structure, further supporting the partial irreversibility of the Ca^2+^ intercalation and the irreversible transformation into CaMn_2_O_4_ as corroborated by the ToF‐SIMS and XPS results.

With progressive cycling, the accumulation of trapped Ca^2+^ in α‐K_0.03_MnO_2_ induces gradual degradation of the tunnel structure. The XRD patterns in Figure S17 (Supporting Information) reveal nearly complete disappear of α‐K_0.03_MnO_2_ diffraction peaks after 10 cycles. Furthermore, HRTEM analysis (Figure S18a,b, Supporting Information) shows no detectable lattice fringes or FFT patterns corresponding to the original α‐K_0.03_MnO_2_ structure. Instead, the nanorods undergo a complete structural transformation into postspinel CaMn_2_O_4_. Note, CaMn_2_O_4_ does not convert back to α‐MnO_2_ upon recharging (Figure S18c,d, Supporting Information), indicating irreversible transformation, which is responsible for the structure degradation.

The gradual formation of CaMn_2_O_4_ from the surface to the bulk of the α‐K_0.03_MnO_2_ particles also reflects the sluggish mobility of Ca^2+^ in the α‐MnO_2_ lattice. To gain deep insights into the diffusion kinetics, DFT calculations were carried out to elucidate the change of the electronic structure and the mobility of Ca^2+^ within α‐MnO_2_. The density of states (DOS) simulation (Figure S19, Supporting Information) reveals that pure α‐MnO_2_ has a bandgap of 1.40 eV, indicating semiconductor behavior. For Ca^2+^ inserted α‐MnO_2_, newly formed occupied states appear, suggesting the coexistence of Mn^3+^ and Mn^4+^ in the structure.^[^ ^26^ ^]^ The bandgap reduces to 0.34 eV, indicating enhanced electronic conductivity. Furthermore, the diffusion paths of the first and the second Ca^2+^ within the 2 × 2 tunnels of α‐MnO_2_ were simulated (Figure 4k,l), representing the intercalation and extraction processes, respectively. Figure 4m shows that the computed activation energies are 0.32 and 0.50 eV for the first and second Ca^2+^ intercalation. The structure models illustrated in Figure 4k,l contain 0.075 and 0.125 Ca^2+^ per formula unit, respectively, significantly lower than that of 0.22 measured by EDS at the discharge state (Figure 3a). Given the increased activation energies (Figure 4m), the diffusion barrier would continually increase upon intercalation of more Ca^2+^. The increased diffusion barrier suggests a more sluggish kinetics in the following de‐/calciation process, which is responsible for the irreversible Ca^2+^ trapping in α‐MnO_2_. The sluggish kinetics of Ca^2+^ induce continuous accumulation of trapped Ca^2+^ within the α‐MnO_2_ framework, while the deep calciation triggers progressive structural transformation into CaMn_2_O_4_. This irreversible phase transition further accelerates capacity fading in the α‐MnO_2_ cathodes during cycling.

Based on the abovementioned findings, the Ca^2+^ insertion mechanism in tunneled α‐MnO_2_ cathodes is summarized in Figure 5b. During the first cycle, Ca^2+^ intercalates into the α‐MnO_2_ structure to form Ca_x_MnO_2_, while the deep calciation induces transformation into CaMn_2_O_4_. With subsequent cycles, continuous Ca^2+^ insertion and irreversible phase transition lead to the complete degradation of the α‐MnO_2_ cathode.

### 2.4 Enhanced Ca^2+^ Storage Performance in the Low‐Crystalline u‐MnO_2_@GO Cathode

To address the sluggish Ca^2+^ diffusion kinetics, ultrasmall MnO_2_ nanoparticles with low crystallinity were synthesized and further combined with graphene oxide (GO) to form u‐MnO_2_@GO composite. The development of disordered structure is a proven strategy to enhance the electrochemical performance of insertion cathodes in various batteries, as it offers more flexible structures for fast ion diffusion.^[^ ^43^ ^]^

**Figure 6:**
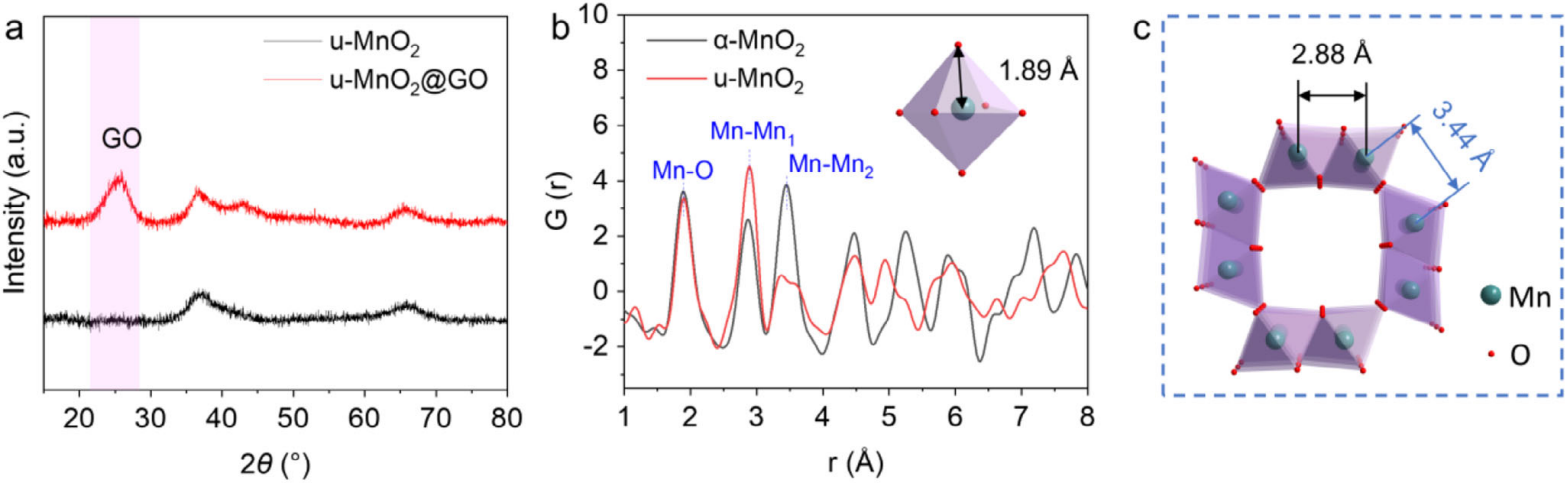
Structural characterization of the as‐synthesized u‐MnO_2_ and u‐MnO_2_@GO samples. a) XRD patterns. b) PDF analysis. c) Schematic illustration of the assembling of [MnO_6_] units for the tunnels of α‐MnO_2_.

As illustrated in **Figure** **6**a, the XRD pattern of u‐MnO_2_ exhibits weak and broad diffraction peaks, demonstrating its low crystallinity. In contrast to the sharp Bragg reflections observed for crystalline α‐MnO_2_, only two broad peaks appear at ≈37° and 67°, corresponding to the strongest diffractions in the standard α‐MnO_2_ pattern. For u‐MnO_2_@GO, the broad peak at 25.3° originates from the (002) plane of GO. TEM image (Figure S20a, Supporting Information) reveals u‐MnO_2_ nanoparticles with a diameter of 10–20 nm anchored on GO sheets, enhancing the electronic conductivity of the composite. HRTEM image (Figure S20b, Supporting Information) reveals long‐range disordered lattices with only short‐range order extending over a few atoms. The corresponding SAED pattern (Figure S20c, Supporting Information) shows no diffraction spots or rings, resulting from the missing of a long‐range ordering structure.

The local structure of u‐MnO_2_ was further probed by X‐ray scattering. The radial distribution accessed by pair distribution function (PDF) analysis (Figure 6b) shows a peak centered at 1.89 Å, corresponding to the Mn─O bond in [MnO_6_] units, which is consistent with that of α‐MnO_2_. This indicates that Mn atoms in the disordered u‐MnO_2_ lattice maintain an octahedral coordination. Peaks at 2.88 and 3.44 Å correspond to Mn─Mn bonds for edge‐sharing and corner‐sharing octahedral [MnO_6_] units, respectively (Figure 6c). Compared to α‐MnO_2_, u‐MnO_2_ exhibits a higher peak intensity at 2.88 Å and a significantly lower intensity at 3.44 Å, suggesting that the edge‐sharing octahedral [MnO_6_] configurations dominate the short‐range structure of u‐MnO_2_.

**Figure 7:**
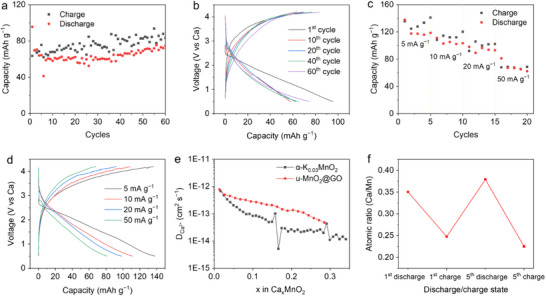
Electrochemical performance of the u‐MnO_2_@GO cathodes in the Ca cells and their comparison with the α‐K_0.03_MnO_2_ cathodes. a) Cycling performance and b) GCD curve of the u‐MnO_2_@GO cathodes at 50 mA g^−1^. Rate performance of the u‐MnO_2_@GO cathode (c) and corresponding GCD curves with increasing current densities (d). e) Diffusion coefficients for the two cathodes. f) EDS results of the u‐MnO_2_@GO cathodes at selected states of charge.

Then, the Ca^2+^ storage performance of u‐MnO_2_@GO cathodes was evaluated in the Ca cells. As shown in **Figure** **7**a,b, the u‐MnO_2_@GO cathode provides a stable capacity of ≈75 mAh g^−1^ at 50 mA g^−1^, with nearly no capacity fades after 60 cycles, demonstrating significantly enhanced cycling stability compared to the α‐K_0.03_MnO_2_ cathode. The higher charge capacity and moderate Coulombic efficiency are likely attributed to degradation of the Ca metal anodes.^[^ ^44^, ^45^ ^]^ The cycle life and capacity retention of u‐MnO_2_@GO cathodes surpass most of the high‐voltage cathodes that are tested against Ca metal anodes, as reported in the literature (Figure 7a; Figure S21, Supporting Information).^[^ ^11^, ^12^, ^46^ ^]^ The rate capability of the u‐MnO_2_@GO cathode was investigated by measuring the discharge–charge capacity at progressively increasing current densities (Figure 7b,c). The u‐MnO_2_@GO cathode achieves average discharge capacities of 121.5, 105.0, 94.1, and 68.5 mAh g^−1^ at 5, 10, 20, and 50 mA g^−1^, respectively. Compared to the α‐K_0.03_MnO_2_ cathode (Figure S22, Supporting Information), the u‐MnO_2_@GO cathode exhibits higher Coulombic efficiency, demonstrating the improved reversibility of Ca^2+^ (de)insertion. The CV curve (Figure S23, Supporting Information) of u‐MnO_2_@GO cathode shows a similar multi‐step reduction process with α‐K_0.03_MnO_2_ cathode, indicating the intercalation of multiple Ca^2+^.

The migration kinetics of Ca^2+^ in the MnO_2_ cathodes were further examined using GITT. As shown in Figure 7e, the Ca^2+^ diffusion coefficient ($D_{Ca^{2+}}$) gradually decreases with the increasing Ca^2+^ content in MnO_2_, particularly for the α‐K_0.03_MnO_2_ cathodes. This suggests that the intercalated Ca^2+^ hinders the subsequent calciation processes, consistent with the DFT results (Figure 4k–m). The $D_{Ca^{2+}}$ values for the u‐MnO_2_@GO cathode are significantly higher than those for the crystallized α‐K_0.03_MnO_2_ cathodes, indicating improved Ca^2+^ migration kinetics in the disordered structure. Therefore, the enhanced electrochemical performance of the u‐MnO_2_@GO cathode compared to the α‐K_0.03_MnO_2_ cathodes can be primarily attributed to the improved Ca^2+^ diffusion within the short‐range ordered framework of u‐MnO_2_. The short‐range ordering structure of u‐MnO_2_ significantly reduces the diffusion distances for Ca^2+^, thereby improving the reaction kinetics at the electrode level. On the other hand, the electronic conductivity of u‐MnO_2_ was enhanced by GO. The long‐range disordered structure of u‐MnO_2_ leads to significantly dispersed energy bands, resulting in inherently lower electronic conductivity. The incorporation of conductive GO effectively improves the charge transport in u‐MnO_2_@GO cathode. In comparison, since the sluggish Ca^2+^ diffusion kinetics is the main cause for the severe capacity fading of α‐K_0.03_MnO_2_ cathode rather than the electrical conductivity, the incorporation of GO does not contribute to the Ca^2+^ storage performance enhancement of α‐K_0.03_MnO_2_ cathode (Figure S24, Supporting Information).

XRD results for the u‐MnO_2_@GO cathodes (Figure S25, Supporting Information) confirm that the structure of u‐MnO_2_ is maintained during discharge/charge, without the formation of new phases. After the first discharge, a high Ca/Mn ratio of 0.35 was observed in EDS results (Figure 7f), suggesting that Ca^2+^ intercalation dominates the charge storage process in the u‐MnO_2_@GO cathodes. Although significant Ca^2+^ trapping within the u‐MnO_2_@GO cathode was also observed after the first cycle, a reversible Ca^2+^ storage of ≈0.15 per formula unit can still be achieved after 5 cycles. The initial Ca^2+^ trapping does not hinder subsequent de‐/calciation process, and the capacity remained almost unchanged with increasing cycles. EDS analysis reveals that the Ca/Mn ratio at the fifth charged state closely matches that of the first charge, indicating no further Ca^2+^ trapping upon cycling. This improved reversibility confirms the enhanced Ca^2+^ migration kinetics in the disordered structure.

## 3 Conclusion

In summary, we carried out a mechanistic study on the charge storage mechanism of α‐K_0.03_MnO_2_ cathodes with 1D tunnel structure. The α‐K_0.03_MnO_2_ cathodes exhibited markedly different electrochemical performance and Ca^2+^ storage capabilities in two distinct cell configurations, utilizing either AC anodes or Ca metal anodes. Structural and elemental analysis revealed that Ca^2+^ insertion/extraction dominates the electrochemical reactions of the α‐K_0.03_MnO_2_ cathodes when cycled against Ca metal anodes, whereas negligible Ca^2+^ intercalation was detected when using AC anodes whether in ether‐ or carbonate‐based electrolytes. While it remains unclear whether this phenomenon applies to other oxide cathodes in the tested electrolytes, this discrepancy underscores the importance of carefully evaluating the charge storage mechanism in Ca‐based systems. It also highlights the necessity of comprehensively probing elemental, structural, and redox changes to provide a complete understanding for the development of truly Ca^2+^ storage cathodes.

On the other hand, the sluggish Ca^2+^ diffusion within the α‐K_0.03_MnO_2_ cathode was identified as a critical issue, leading to irreversible Ca^2+^ trapping and severe capacity fading. More importantly, progressive Ca^2+^ accumulation induces deep calciation, triggering irreversible transformation into CaMn_2_O_4_ and eventually complete structural collapse. Targeting this issue, we developed a disordered u‐MnO_2_@GO cathode, which demonstrated significantly enhanced cycling stability over 60 cycles and superior rate performance (up to 50 mA g^−1^), benefiting from the improved Ca^2+^ diffusion kinetics. Our work provides new insights into the Ca^2+^ storage mechanism and showcases effective strategies for enhancing Ca^2+^ diffusion. We hope this study inspires further advancements in the development of high‐voltage cathodes for Ca batteries.

## Conflict of Interest

The authors declare no conflict of interest.

## Supporting information



Supporting Information

## Data Availability

The data that support the findings of this study are available from the corresponding author upon reasonable request.

## References

[1] Y. Tian , G. Zeng , A. Rutt , T. Shi , H. Kim , J. Wang , J. Koettgen , Y. Sun , B. Ouyang , T. Chen , Z. Lun , Z. Rong , K. Persson , G. Ceder , Chem. Rev. 2021, 121, 1623. 33356176 10.1021/acs.chemrev.0c00767

[2] M. E. Arroyo‐de Dompablo , A. Ponrouch , P. Johansson , M. R. Palacín , Chem. Rev. 2020, 120, 6331. 31661250 10.1021/acs.chemrev.9b00339

[3] Y. L. Liang , H. Dong , D. Aurbach , Y. Yao , Nat. Energy 2020, 5, 646.

[4] J. Kim , M. Kim , J. Lee , J. An , S. Yang , H. C. Ahn , D. J. Yoo , J. W. Choi , Chem. Soc. Rev. 2024, 53, 8878. 39106108 10.1039/d4cs00557k

[5] D. S. Tchitchekova , A. Ponrouch , R. Verrelli , T. Broux , C. Frontera , A. Sorrentino , F. Bardé , N. Biskup , M. E. Arroyo‐de Dompablo , M. R. Palacín , Chem. Mater. 2018, 30, 847.

[6] Z. Y. Li , S. S. Cui , J. Häcker , M. Nojabaee , M. Fichtner , G. L. Cui , Z. Zhao‐Karger , Angew. Chem., Int. Ed. 2025, 64, 202415942. 10.1002/anie.202415942 39679638

[7] H. R. Tinker , M. Li , A. P. Vijaya Kumar Saroja , Y. Liu , Y. Luo , W. Ren , R. Wei , Y. Lu , P. He , Y. Han , C. A. Howard , F. Corà , Y. Xu , Small Struct. 2024, 5, 2400317.

[8] A. L. Lipson , B. F. Pan , S. H. Lapidus , C. Liao , J. T. Vaughey , B. J. Ingram , Chem. Mater. 2015, 27, 8442.

[9] B. Jeon , J. W. Heo , J. Hyoung , H. H. Kwak , D. M. Lee , S.‐T. Hong , Chem. Mater. 2020, 32, 8772.

[10] C. Chen , F. Shi , S. Zhang , Y. Su , Z. L. Xu , Small 2022, 18, 2107853. 10.1002/smll.202107853 35388645

[11] S. Kim , L. Yin , M. H. Lee , P. Parajuli , L. Blanc , T. T. Fister , H. Park , B. J. Kwon , B. J. Ingram , P. Zapol , R. F. Klie , K. Kang , L. F. Nazar , S. H. Lapidus , J. T. Vaughey , ACS Energy Lett. 2020, 5, 3203.

[12] Z. L. Xu , J. Park , J. Wang , H. Moon , G. Yoon , J. Lim , Y. J. Ko , S. P. Cho , S. Y. Lee , K. Kang , Nat. Commun. 2021, 12, 3369. 34099694 10.1038/s41467-021-23703-x PMC8184813

[13] X. Xu , M. Duan , Y. Yue , Q. Li , X. Zhang , L. Wu , P. Wu , B. Song , L. Mai , ACS Energy Lett. 2019, 4, 1328.

[14] M. Cabello , F. Nacimiento , R. Alcántara , P. Lavela , C. Pérez Vicente , J. L. Tirado , Chem. Mater. 2018, 30, 5853. 10.3390/nano8070501 PMC6071289 29986454

[15] H. Bu , H. Lee , J. Hyoung , J. W. Heo , D. Kim , Y. J. Lee , S.‐T. Hong , Chem. Mater. 2023, 35, 7974.

[16] K. Kisu , R. Mohtadi , S. I. Orimo , Adv. Sci. 2023, 10, 2301178. 10.1002/advs.202301178 PMC10401151 37208795

[17] Z. Li , B. P. Vinayan , P. Jankowski , C. Njel , A. Roy , T. Vegge , J. Maibach , J. M. G. Lastra , M. Fichtner , Z. Zhao‐Karger , Angew. Chem., Int. Ed. 2020, 59, 11483. 10.1002/anie.202002560 PMC7384178 32220137

[18] X. Guo , S. Yang , D. Wang , A. Chen , Y. Wang , P. Li , G. Liang , C. Zhi , Curr. Opin. Electrochem. 2021, 30, 100769.

[19] Q. H. Zhao , A. Y. Song , S. X. Ding , R. Z. Qin , Y. H. Cui , S. N. Li , F. Pan , Adv. Mater. 2020, 32, 2002450. 10.1002/adma.202002450 33165987

[20] C. Zuo , F. Xiong , J. Wang , Y. An , L. Zhang , Q. An , Adv. Funct. Mater. 2022, 32, 2202975.

[21] S. Y. Hou , X. Ji , K. Gaskell , P. F. Wang , L. N. Wang , J. J. Xu , R. M. Sun , O. Borodin , C. S. Wang , Science 2021, 374, 172. 34618574 10.1126/science.abg3954

[22] J. D. Forero‐Saboya , E. Marchante , R. B. Araujo , D. Monti , P. Johansson , A. Ponrouch , J. Phys. Chem. C 2019, 123, 29524. 10.1021/acs.jpcc.9b07308 PMC6961307 31956392

[23] A. Du , Z. Li , M. Fichtner , G. Cui , Green Carbon 2025, 10.1016/j.greenca.2025.03.002.

[24] R. Verrelli , A. P. Black , C. Pattanathummasid , D. S. Tchitchekova , A. Ponrouch , J. Oró‐Solé , C. Frontera , F. Bardé , P. Rozier , M. R. Palacín , J. Power Sources 2018, 407, 162.

[25] M. J. Young , A. M. Holder , S. M. George , C. B. Musgrave , Chem. Mater. 2015, 27, 1172.

[26] Y. Yuan , C. Zhan , K. He , H. Chen , W. Yao , S. Sharifi‐Asl , B. Song , Z. Yang , A. Nie , X. Luo , H. Wang , S. M. Wood , K. Amine , M. S. Islam , J. Lu , R. Shahbazian‐Yassar , Nat. Commun. 2016, 7, 13374. 27869120 10.1038/ncomms13374 PMC5473628

[27] T. Gao , M. Glerup , F. Krumeich , R. Nesper , H. Fjellvåg , P. Norby , J. Phys. Chem. C 2008, 112, 13134.

[28] Z. Yang , D. C. Ford , J. S. Park , Y. Ren , S. Kim , H. Kim , T. T. Fister , M. K. Y. Chan , M. M. Thackeray , Chem. Mater. 2017, 29, 1507.

[29] T. Gao , H. Fjellvag , P. Norby , Anal. Chim. Acta 2009, 648, 235. 19646589 10.1016/j.aca.2009.06.059

[30] T. R. Juran , J. Young , M. Smeu , J. Phys. Chem. C 2018, 122, 8788.

[31] S. Cheng , L. F. Yang , D. C. Chen , X. Ji , Z. J. Jiang , D. Ding , M. L. Liu , Nano Energy 2014, 9, 161.

[32] G. Fang , C. Zhu , M. Chen , J. Zhou , B. Tang , X. Cao , X. Zheng , A. Pan , S. Liang , Adv. Funct. Mater. 2019, 29, 1808375.

[33] L. Ye , M. Liao , K. Zhang , M. Zheng , Y. Jiang , X. Cheng , C. Wang , Q. Xu , C. Tang , P. Li , Y. Wen , Y. Xu , X. Sun , P. Chen , H. Sun , Y. Gao , Y. Zhang , B. Wang , J. Lu , H. Zhou , Y. Wang , Y. Xia , X. Xu , H. Peng , Nature 2024, 626, 313. 38326591 10.1038/s41586-023-06949-x

[34] X. H. Yu , Z. Wen , H. L. Li , S. T. Tu , J. Y. Yan , Fuel 2011, 90, 1868.

[35] D. A. Michalski , C. Wickleder , P. M. Kaul , Propell. Explos. Pyrot. 2025, 50, 202400214.

[36] N. N. Rajput , T. J. Seguin , B. M. Wood , X. H. Qu , K. A. Persson , Top. Curr. Chem. 2018, 376, 19. 10.1007/s41061-018-0195-2 PMC5920006 29700688

[37] G. Wan , T. P. Pollard , L. Ma , M. A. Schroeder , C. C. Chen , Z. Zhu , Z. Zhang , C. J. Sun , J. Cai , H. L. Thaman , A. Vailionis , H. Li , S. Kelly , Z. Feng , J. Franklin , S. P. Harvey , Y. Zhang , Y. Du , Z. Chen , C. J. Tassone , H. G. Steinruck , K. Xu , O. Borodin , M. F. Toney , Science 2024, 385, 1230. 39265020 10.1126/science.adg4687

[38] Y. F. Yuan , A. M. Nie , G. M. Odegard , R. Xu , D. H. Zhou , S. Santhanagopalan , K. He , H. Asayesh‐Ardakani , D. D. Meng , R. F. Klie , C. Johnson , J. Lu , R. Shahbazian‐Yassar , Nano Lett. 2015, 15, 2998. 25871572 10.1021/nl5048913

[39] Y. F. Yuan , L. Ma , K. He , W. T. Yao , A. Nie , X. X. Bi , K. Amine , T. P. Wu , J. Lu , R. Shahbazian‐Yassr , Nano Energy 2016, 19, 382.

[40] D. A. Tompsett , M. S. Islam , Chem. Mater. 2013, 25, 2515.

[41] T. S. Arthur , R. Zhang , C. Ling , P. A. Glans , X. Fan , J. Guo , F. Mizuno , ACS Appl. Mater. Interfaces 2014, 6, 7004. 24807043 10.1021/am5015327

[42] M. E. A.‐d. Dompablo , C. Krich , J. Nava‐Avendaño , N. Biškup , M. R. Palacín , F. Bardé , Chem. Mater. 2016, 28, 6886. 10.1039/c6cp03381d 27398629

[43] L. Zhang , L. Yu , O. L. Li , S.‐Y. Choi , G. Saeed , D. Lee , K. H. Kim , ACS Appl. Energy Mater. 2022, 5, 5954.

[44] Z. Zhao‐Karger , Y. Xiu , Z. Li , A. Reupert , T. Smok , M. Fichtner , Nat. Commun. 2022, 13, 3849. 35788588 10.1038/s41467-022-31261-z PMC9253317

[45] J. Bitenc , A. Scafuri , K. Pirnat , M. Lozinšek , I. Jerman , J. Grdadolnik , B. Fraisse , R. Berthelot , L. Stievano , R. Dominko , Batteries Supercaps 2021, 4, 214.

[46] K. V. Nielson , J. Luo , T. L. Liu , Batteries Supercaps 2020, 3, 766.

