# Supplementary material for: Calcium‐Ion Insertion Chemistry in Tunneled α‐MnO2 Cathodes for Calcium Metal Batteries
Source: Adv Sci (Weinh). 2025 Sep 12;12(44):e08050. doi: 10.1002/advs.202508050 (PMC12667528; doi:10.1002/advs.202508050)
Supplement: Supplementary file 1 — Supporting Information [file ADVS-12-e08050-s001.docx]

Supporting information

**Ca-Ion Insertion Chemistry in Tunneled α-MnO_2_ Cathodes for Ca Metal Batteries**

Shuangshuang Cui,^[a]^ Yang Wang,^[a]^ Dechen Zeng,^[a]^ Aobing Du,^[a]^ Zhaolin Lv,^[a]^ Andi Wang,^[b]^ Zhenyou Li,*^[a]^ Guanglei Cui*^[a]^

[a] Dr. S. Cui, Y. Wang, D. Zeng, Prof. A. Du, Dr. Z. Lv, Prof. Z. Li,* Prof. G. Cui*
Qingdao New Energy Shandong Laboratory

Qingdao Institute of Bioenergy and Bioprocess Technology
Chinese Academy of Sciences
No. 189 Songling Road, Laoshan District, Qingdao, Shandong 266101, China
E-mail: [lizhenyou@qibebt.ac.cn](mailto:lizhenyou@qibebt.ac.cn); [cuigl@qibebt.ac.cn](mailto:cuigl@qibebt.ac.cn)

[b] A. Wang
Analytical and Testing Center, South China University of Technology
No. 381 Wushan Road, Tianhe District, Guangzhou, Guangdong 510640, China

**Experimental section**

*Synthesis of α-MnO_2_*

α-MnO_2_ was synthesized using a previously reported hydrothermal method.^[1]^ In a typical procedure, 6 mmol of KMnO_4_ was dissolved in 70 mL of deionized water. Subsequently, 3 mL of concentrated H_2_SO_4_ was added into the KMnO_4_ solution under vigorous stirring. The solution was transferred to a Teflon-lined autoclave and heated to 150 °C, where it was maintained for 24 h. After the reaction, the solid products were collected through centrifugation, washed multiple times with deionized water, and dried at 80 °C for 12 h.

*Synthesis of K^+^-deficient α-K_0.03_MnO_2_*

The obtained α-MnO_2_ contained a residual amount of K^+^ within its tunneled structure. To remove these K^+^, an acid treatment using concentrated HNO_3_ was employed.^[2]^ Specifically, the α-MnO_2_ powder was added to concentrated HNO_3_ and the mixture was stirred continuously for 5 days. After the treatment, the products were collected through centrifugation and thoroughly washed with deionized water until the upper solution reached a neutral pH (pH≈7). Finally, the precipitate was dried at 80 °C for 12 h.

*Synthesis of u-MnO_2_@GO*

The u-MnO_2_@GO composite was synthesized through a reaction between KMnO_4_ and ethanol at room temperature. Firstly, 0.2 g of graphene oxide (GO) was dispersed in 25 mL of H_2_O using ultrasonication for 30 min. Then, 0.5 g of KMnO_4_ was added to the GO suspension under vigorous stirring for 30 min. Finally, 50 mL of ethanol was introduced into the mixture and stirring for another 2 h. The resulting brown products were collected through centrifugation, washed multiple times with deionized water, and dried at 80 °C for 12 h. For comparison, u-MnO_2_ was synthesized using the same method without the addition of GO.

*Fabrication of α-K_0.03_MnO_2_@GO composite*

Firstly, 0.1 g of GO was dispersed in 100 mL of H_2_O using ultrasonication for 30 min. Then, 0.3 g of α-K_0.03_MnO_2_ was added to the GO suspension and sonicated for another 2 h. The resulting products of α-K_0.03_MnO_2_@GO were collected through centrifugation and dried at 80 °C for 12 h.

*Characterizations*

X-ray diffraction (XRD) analysis were conducted using a Rigaku SmartLab diffractometer with Cu Kα radiation (λ=1.5406 Å). XRD patterns were recorded at a scanning rate of 5° min^−1^. To probe the local structure of the materials, X-ray scattering measurements were performed using another Rigaku SmartLab diffractometer with Ag Kα radiation (λ= 0.5594 Å). The morphology of α-MnO_2_ powder and electrodes was characterized by field-emission scanning electron microscopy (FE-SEM, Zeiss G-360). SEM images were acquired using an InLens detector at an acceleration voltage of 15 kV and a working distance of 8–10 mm. Elemental compositions of the as-synthesized samples and cathodes were measured by inductively coupled plasma optical emission spectroscopy (ICP-OES, PerkinElmer Optima 2100DV) and energy-dispersive X-ray spectroscopy (EDS). Chemical bonding and structural features were further investigated using Raman spectroscopy (Thermo Scientific DXR Raman microscope) and X-ray photoelectron spectroscopy (XPS, Shimadzu AXIS SUPRA+) with monochromatic Al Kα (1486.6 eV) radiation. The binding energy (BE) of XPS spectra was charge referenced to the C 1s peak at 284.8 eV. Transmission electron microscopy (TEM) measurements were performed on a FEI Talos F200X microscope operated at 200 kV. Time-of-flight secondary-ion mass spectrometry (ToF-SIMS) measurement was taken using ToF-SIMS M6 (IONTOF).

*Electrochemical Measurements*

The cathode slurry was prepared by mixing α-MnO_2_, conductive carbon black (Super P) and polyvinylidene fluoride (PVDF) in a weight ratio of 7:2:1 in N-methylpyrrolidone (NMP). The slurry was coated on an aluminium foil and vacuum dried at 80 °C overnight. The mass loading of α-MnO_2_ on the electrode is 0.8–1.2 mg cm^−2^. CR2032 coin cells were assembled using the α-MnO_2_ electrode as the cathode, a Ca pellet as the anode, a glass fibre separator (Whatman GF/D), and a 0.25 M calcium tetrakis(hexafluoroisopropyloxy)borate (Ca[B(hfip)_4_]_2_) in dimethoxyethane (DME) as the electrolyte. The Ca[B(hfip)_4_]_2_ was synthesized following a previous report.^[3]^ For comparison, the α-MnO_2_ cathodes were also investigated in model cell configurations using an activated carbon (AC) anode and a 0.5 M calcium bis(trifluoromethane sulfonyl)imide (Ca(TFSI)_2_)/DME electrolyte. The Ca(TFSI)_2_ salts were dried at 120 °C under vacuum for 48 h before use. DME was dried using 3 Å molecular sieves for more than 24 h before use. The water content of solvent was confirmed to be less than 20 ppm via Karl Fisher titration. The carbonate-based solvents including ethylene carbonate (EC), propylene carbonate (PC), dimethyl carbonate (DMC) and ethyl methyl carbonate (EMC) used in this work were treated with the same way. The AC anodes were fabricated using activated carbon (AC) powders (Kuraray, YP-80F) and polytetrafluoroethylene (PTFE) binder. The AC powders consist primarily of carbon, with trace amounts of Fe (7 ppm) and Cu (22 ppm), and contain no detectable Ca. The anode preparation involved mixing AC powders with PTFE emulsion in an 8:2 weight ratio using ethanol as the dispersion medium. The homogeneous mixture was then rolled into a thick film with uniform thickness and dried at 80 °C for 8 hours. Circular electrodes (12 mm diameter) were punched from the dried films, achieving a mass loading of approximately 40 mg cm^−2^. Before use, the AC anodes were dried at 120 °C under vacuum for 48 h to remove the adsorbed H_2_O on AC.

Galvanostatic charge/discharge tests were performed using a battery testing system (LAND, Wuhan) at 30 °C. Cyclic voltammetry (CV) measurements were conducted using an electrochemical workstation (Corrtest CS310X). Galvanostatic intermittent titration technique (GITT) measurements were carried out by applying a negative current pulse of 10 mA g^−1^ for 30 min, followed by a relaxation of 2 h. This sequence was repeated until the cell was fully discharged and the voltage reached the lower cutoff. The chemical diffusion coefficient of Ca ions ($\text{D}_{\text{Ca}^{\text{2+}}}$) within the cathode was calculated using the following equation,

$\text{D}_{\text{Ca}^{\text{2+}}}$ =$\text{ }\frac{\text{4}}{\text{πτ}}$($\frac{\text{n}_{\text{m}}\text{V}_{\text{m}}}{\text{S}}$)^2^($\frac{\text{ΔE}_{\text{s}}}{\text{ΔE}_{\text{t}}}$)^2^

where τ is the relaxation time, n_m_ and V_m_ are the moles and molar volume of the active material, respectively, S is the surface area of the cathode, ΔE_s_ and ΔE_t_ is the voltage change during the discharge/charge pulse, and the relaxation process, respectively.

*DFT calculations*

Density functional theory (DFT) calculations were performed using first-principles methods^[4-5]^ within the generalized gradient approximation (GGA) employing the Perdew-Burke-Ernzerhof (PBE)^[6]^ formulation. The projected augmented wave (PAW) potentials^[7-8]^ were used to describe the ionic cores, with valence electrons represented using a plane wave basis set with a kinetic energy cutoff of 520 eV. Partial occupancies of the Kohn−Sham orbitals were treated using the Gaussian smearing method with a width of 0.05 eV. The electronic energy was considered self-consistent when the energy change was smaller than 10^−5^ eV. Geometry optimization was considered convergent when the energy change was less than 0.05 eV Å^−1^. For the α-MnO_2_ structure, the U correction is used for Mn (3.9 eV) atoms. Brillouin zone integration was performed using 2×2×1 Monkhorst-Pack k-point sampling for a structure. The Ca^2+^ diffusion pathways and energy barriers were determined using the nudged elastic band (NEB) method, with the transition state of an elementary reaction step. In the NEB method, the path between the reactant(s) and product(s) was discretized into a series of intermediate structural images. These images were relaxed until the perpendicular forces were reduced to below 0.03 eV Å^−1^.


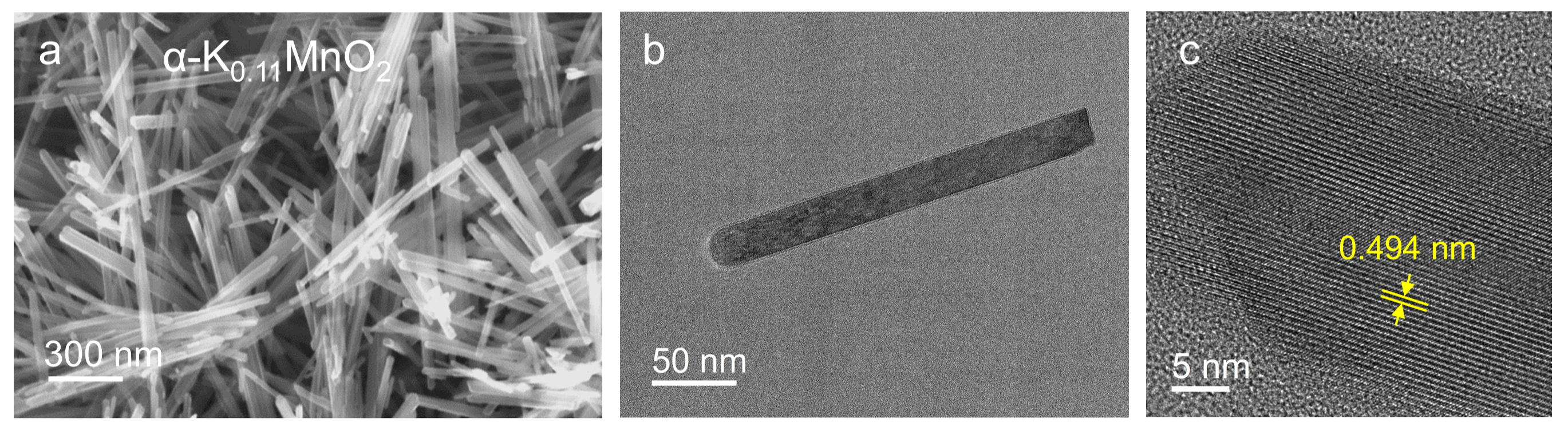


**Figure. S1** Morphological characterization of α-K_0.11_MnO_2_. (a) SEM image; (b) TEM image; (c) HRTEM image.


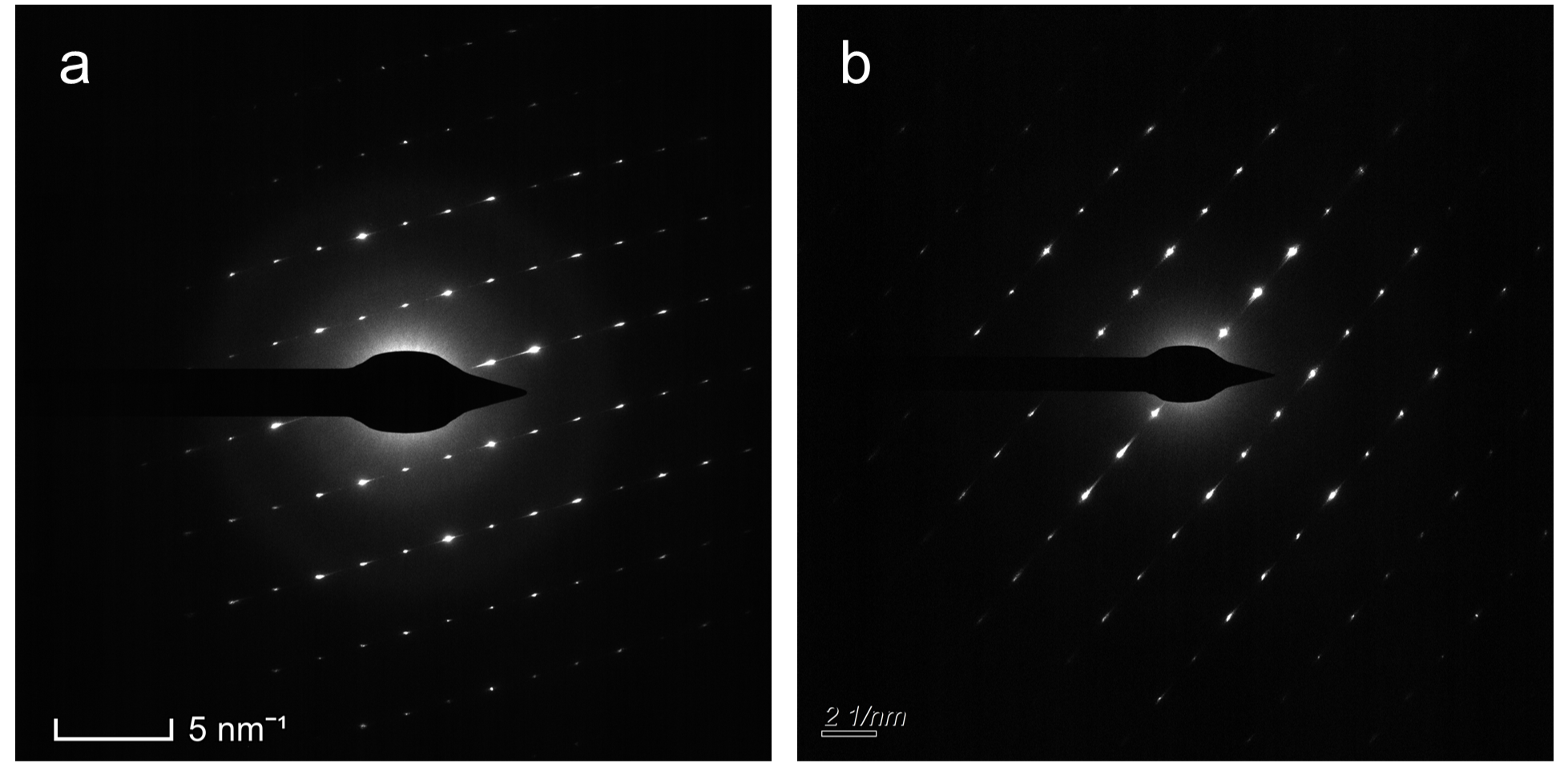


**Figure. S2** Selected area electron diﬀraction (SAED) pattern of (a) α-K_0.11_MnO_2_, (b) α-K_0.03_MnO_2_.


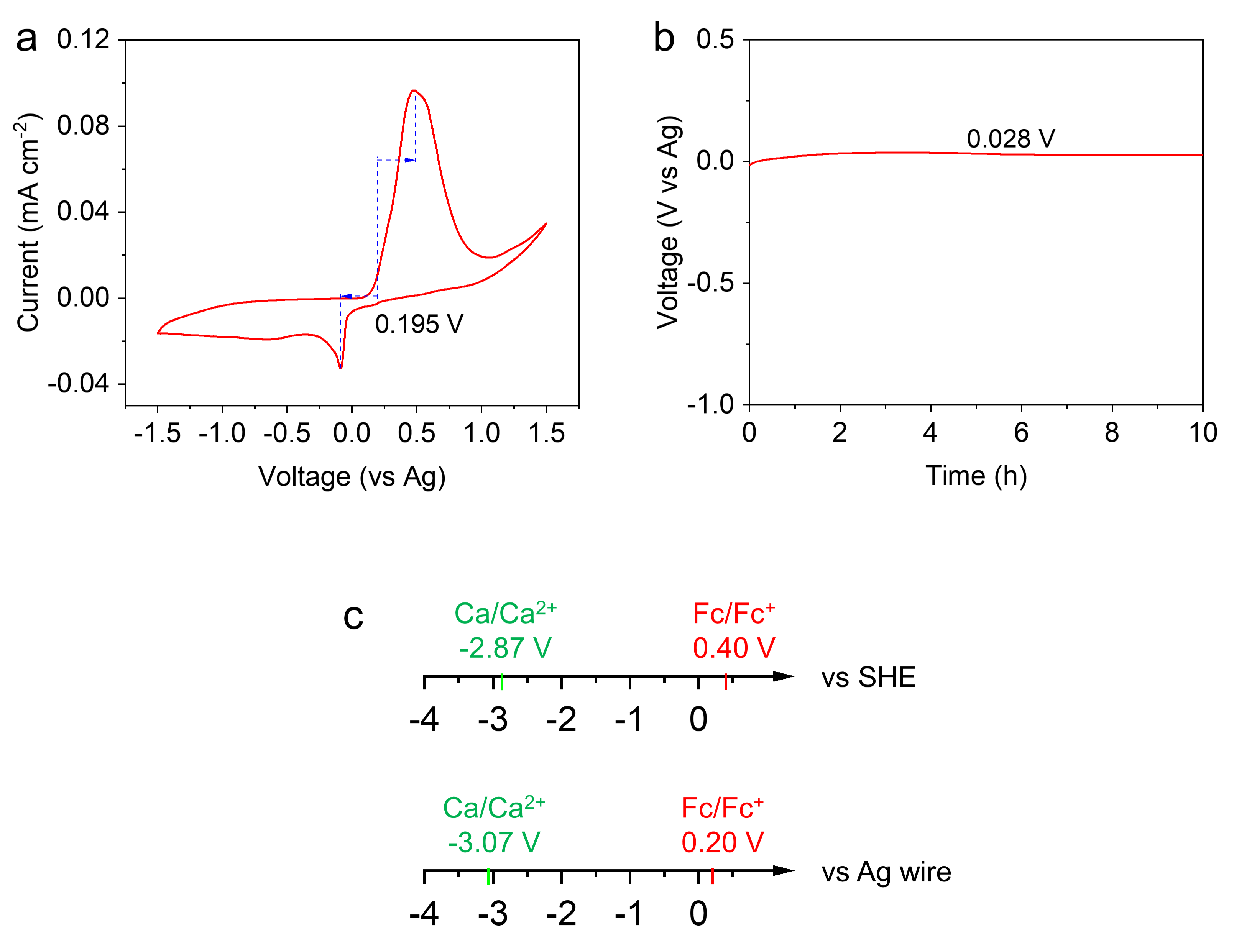


**Figure. S3** (a) CV test of 5 mM ferrocene dissolved in 0.5 M Ca(TFSI)_2_/DME electrolyte (the working and counter electrodes are Pt and Au plate, the reference electrode is Ag wire) at a scan rate of 0.2 mV s^−1^. The Fc^+^/Fc redox couple is measured at about 0.195 V vs. Ag wire; (b) Open circuit voltage (OCV) of AC vs. Ag wire reference electrode in 0.5 M Ca(TFSI)_2_/DME; (c) The potential of internal reference Fc/Fc^+^ vs. SHE is 0.40 V and the OCV of AC vs. Ag wire is 0.028 V. Therefore, the Ag wire reference voltage can be estimated to be 3.07 V vs. Ca^2+^/Ca and the AC voltage can be estimated to be about 3.098 V vs. Ca^2+^/Ca.


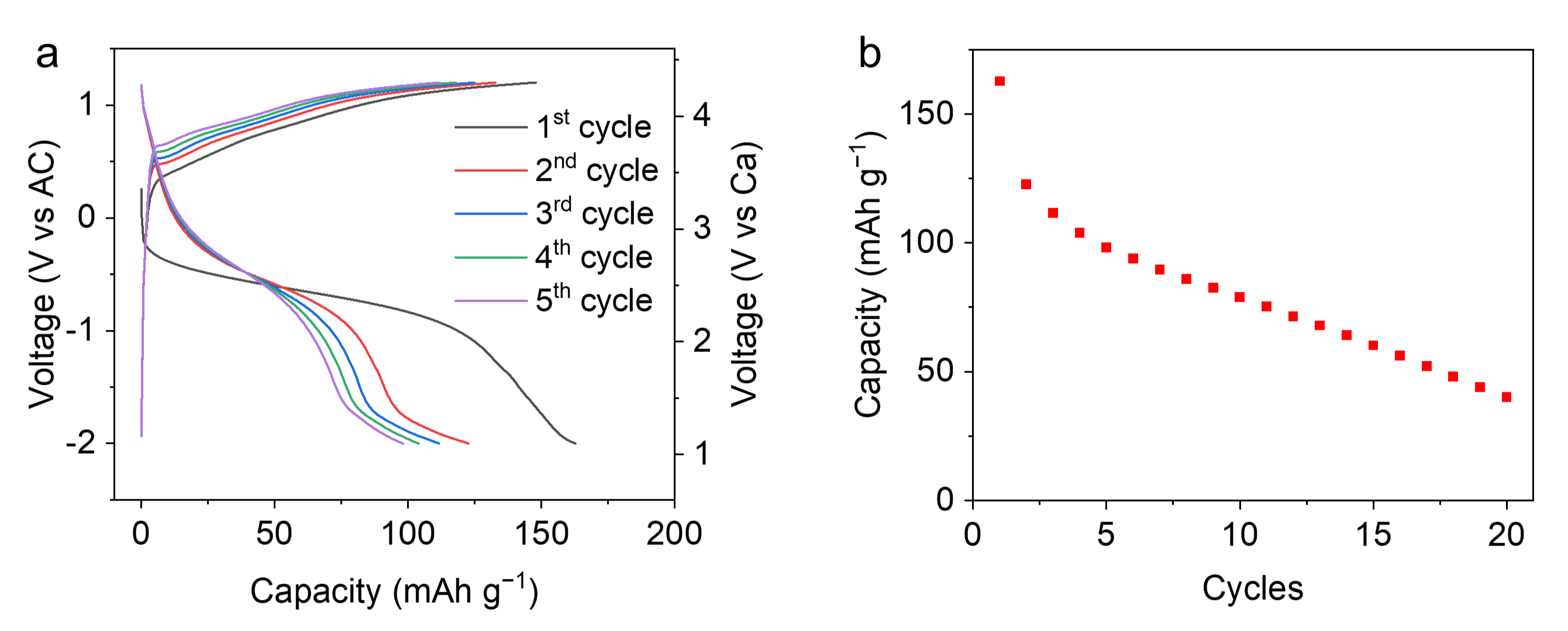


**Figure. S4** Electrochemical performance of α-K_0.03_MnO_2_ cathode at a current density of 50 mA g^−1^ in the cells with an AC anode within the voltage range between −2.0 V and 1.2 V vs. AC. (a) GCD curves; (b) Cycling performance.


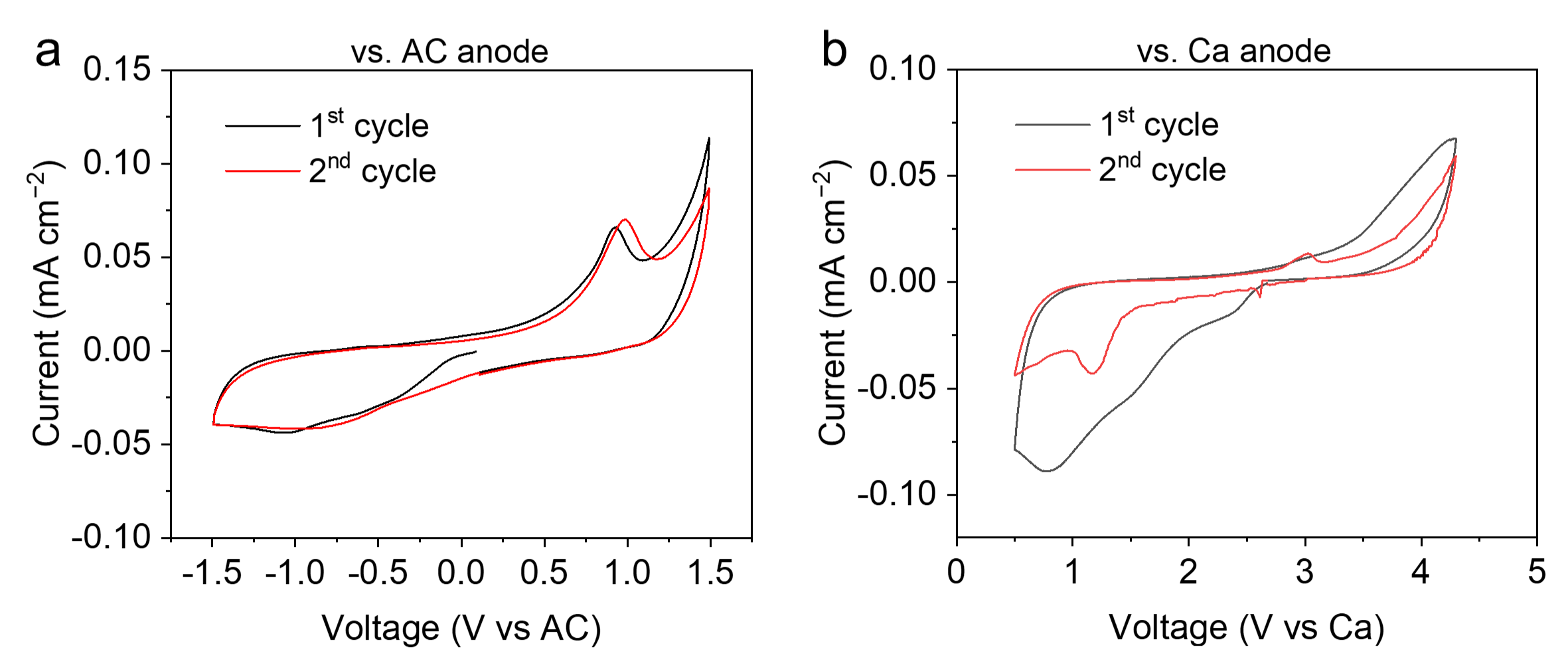


**Figure. S5** CV curves of the α-K_0.03_MnO_2_ cathode at a scan rate of 0.2 mV s^−1^ in the cells with an AC anode (a), or with a Ca anode (b).


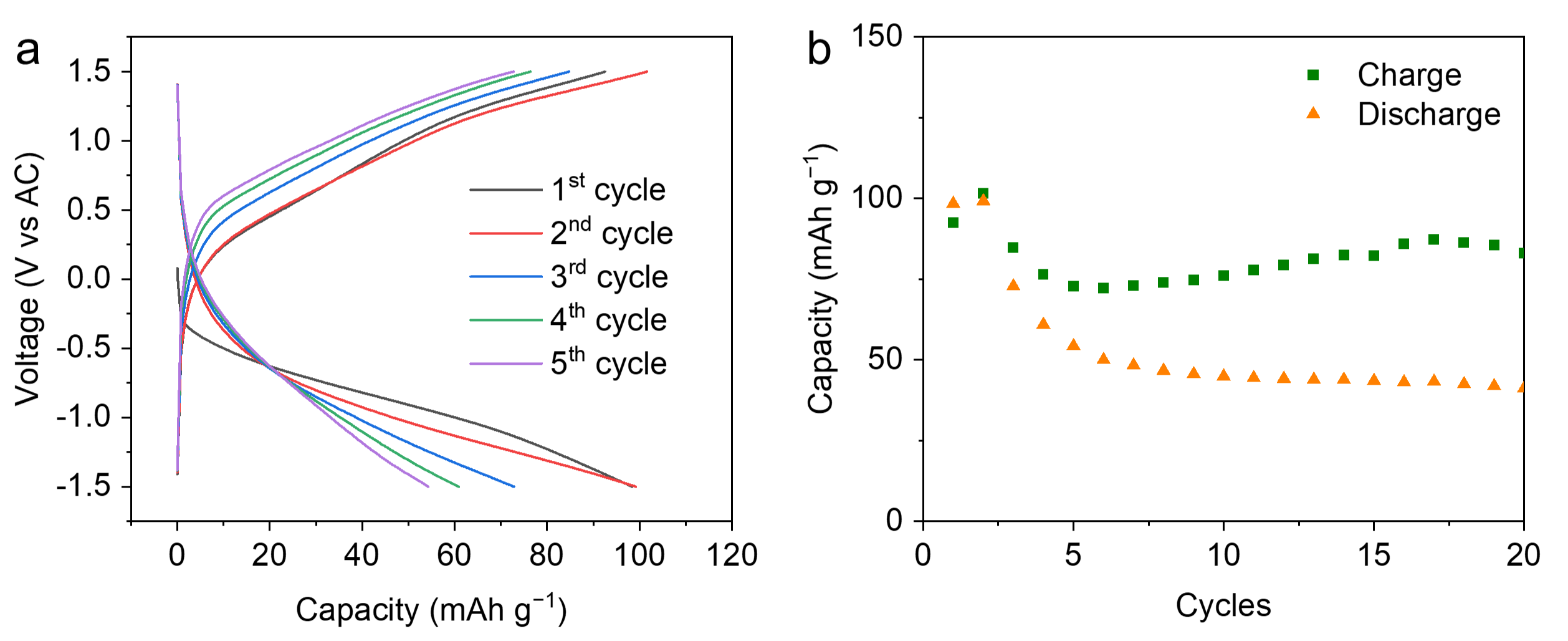


**Figure. S6** Electrochemical performance of the α-K_0.03_MnO_2_ cathode vs. AC anode and in the electrolyte of 0.25 M Ca[B(hfip)_4_]_2_ in DME at a current density of 50 mA g^−1^. (a) GCD curves, (b) cycling performance.


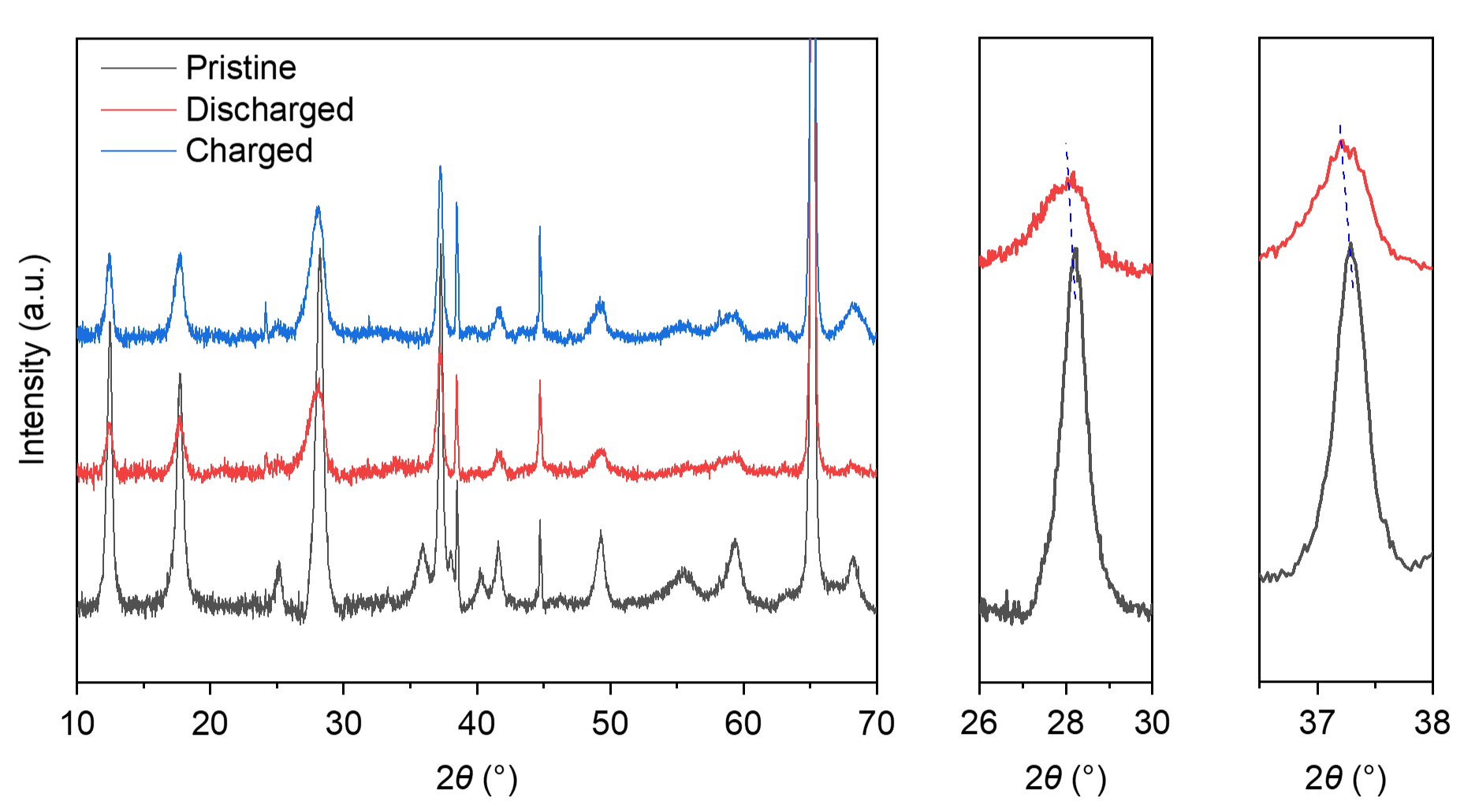


**Figure. S7** *Ex situ* XRD patterns of the α-K_0.03_MnO_2_ cathode during the first cycle with AC anode and 0.5 M Ca(TFSI)_2_-DME electrolyte.

**Figure. S8** EDS results for the K^+^ content and the corresponding GCD curve.


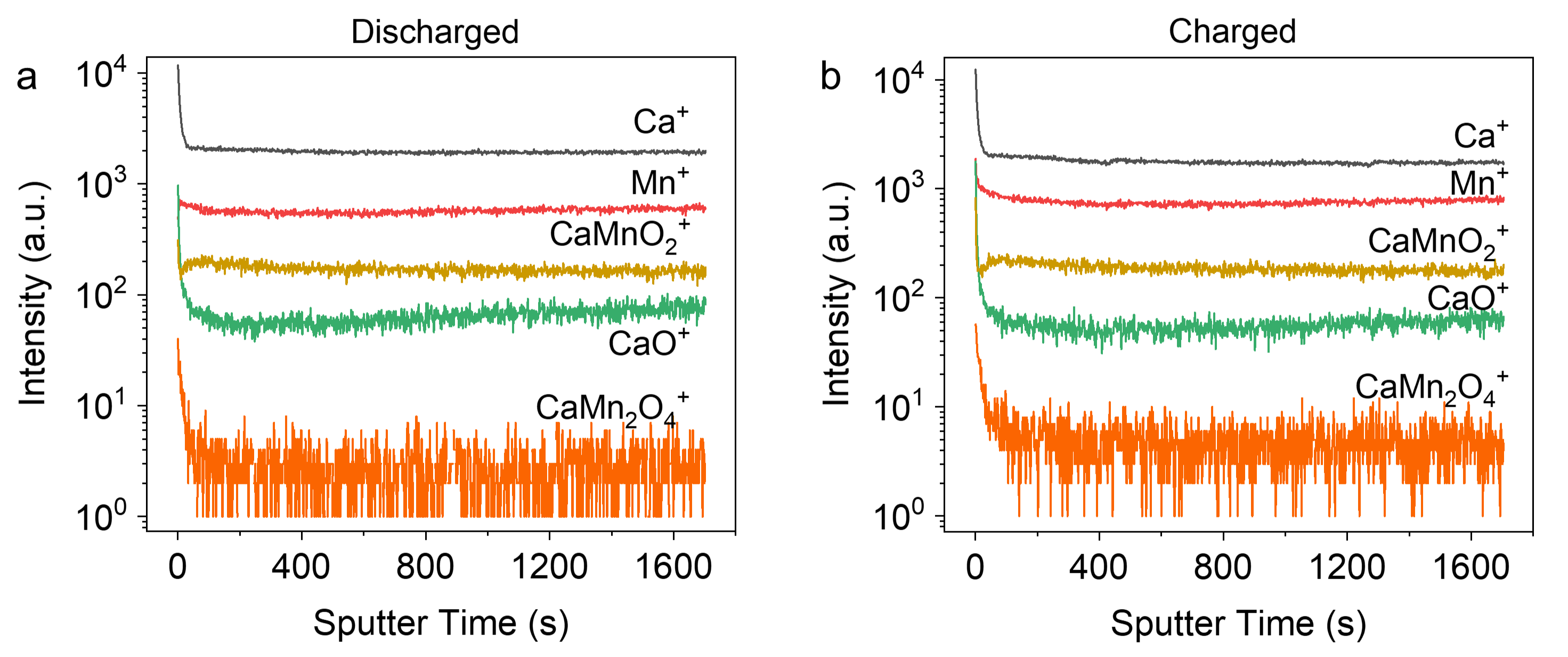


**Figure. S9** TOF-SIMS depth profile of representative secondary ion species obtained by sputtering the discharged cathode (a), charged cathode (b).

**Figure. S10** Raman spectra of the α-K_0.03_MnO_2_ cathode during the first discharge/charge.


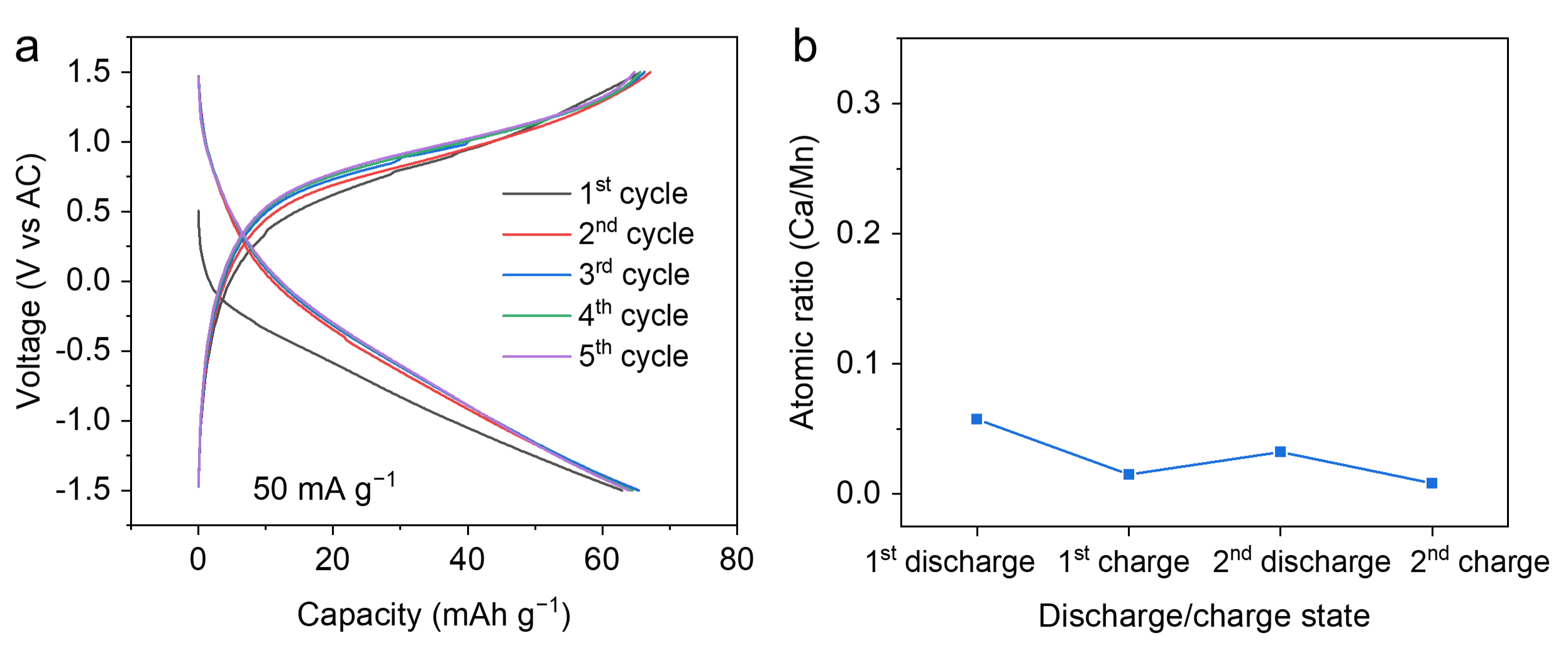


**Figure. S11** Electrochemical performance and the elemental analysis of the α-K_0.03_MnO_2_ cathode in the AC cells, with a 0.8 M Ca(TFSI)_2_-EC/PC/DMC/EMC (v/v, 3:3:2:2) electrolyte. (a) GCD curves; (b) EDS results.

**Figure. S12** Diﬀusion coeﬃcients for the α-K_0.03_MnO_2_ cathode in the cells with an AC anode or with a Ca anode.


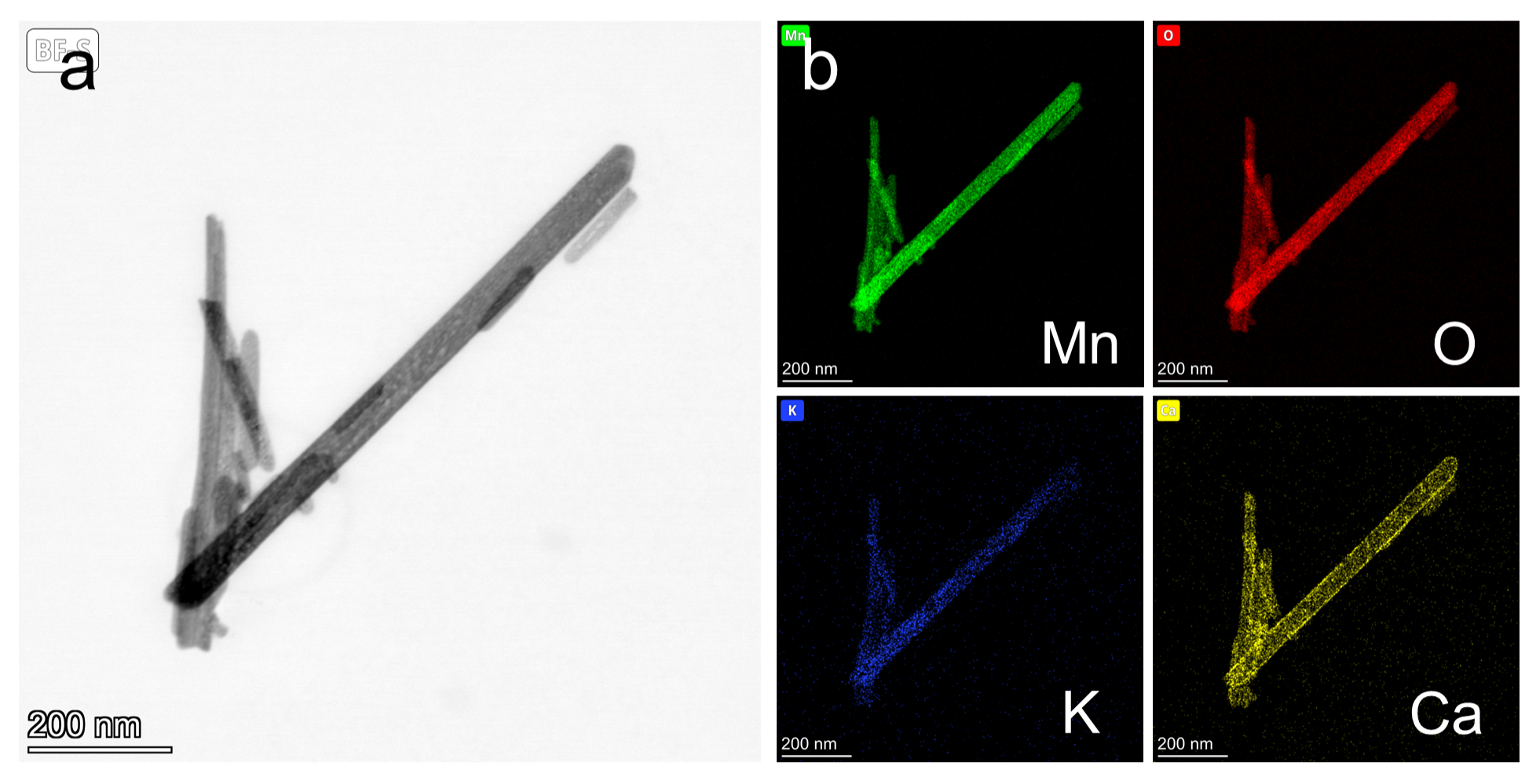


**Figure. S13** (a) TEM image, (b) elemental mapping images of the α-K_0.03_MnO_2_ cathode at the discharged state in the Ca cells.


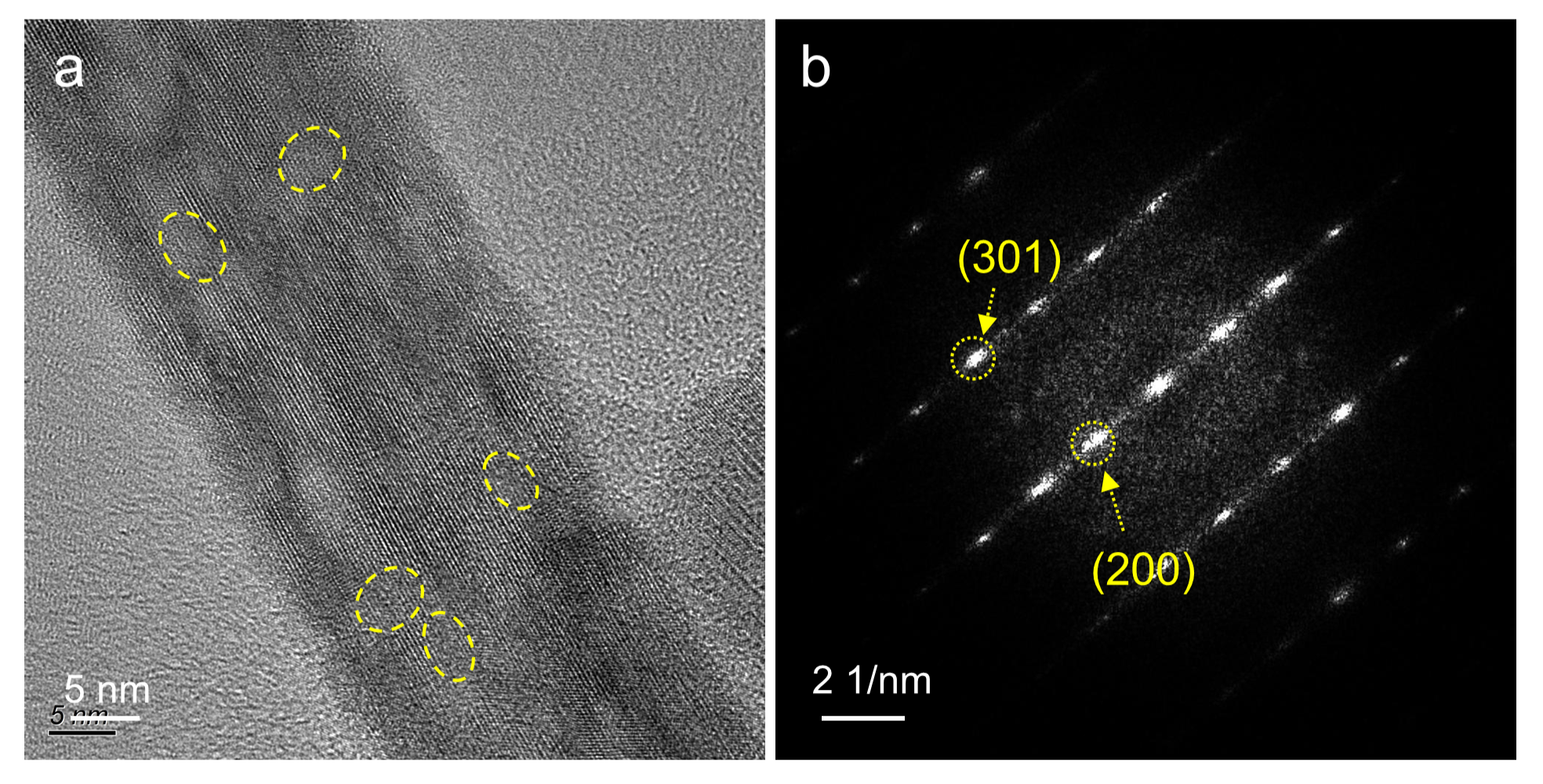


**Figure. S14** (a) HRTEM image, (b) FFT pattern of the α-K_0.03_MnO_2_ cathode at the discharged state with AC anodes and 0.5 M Ca(TFSI)_2_-DME electrolyte.


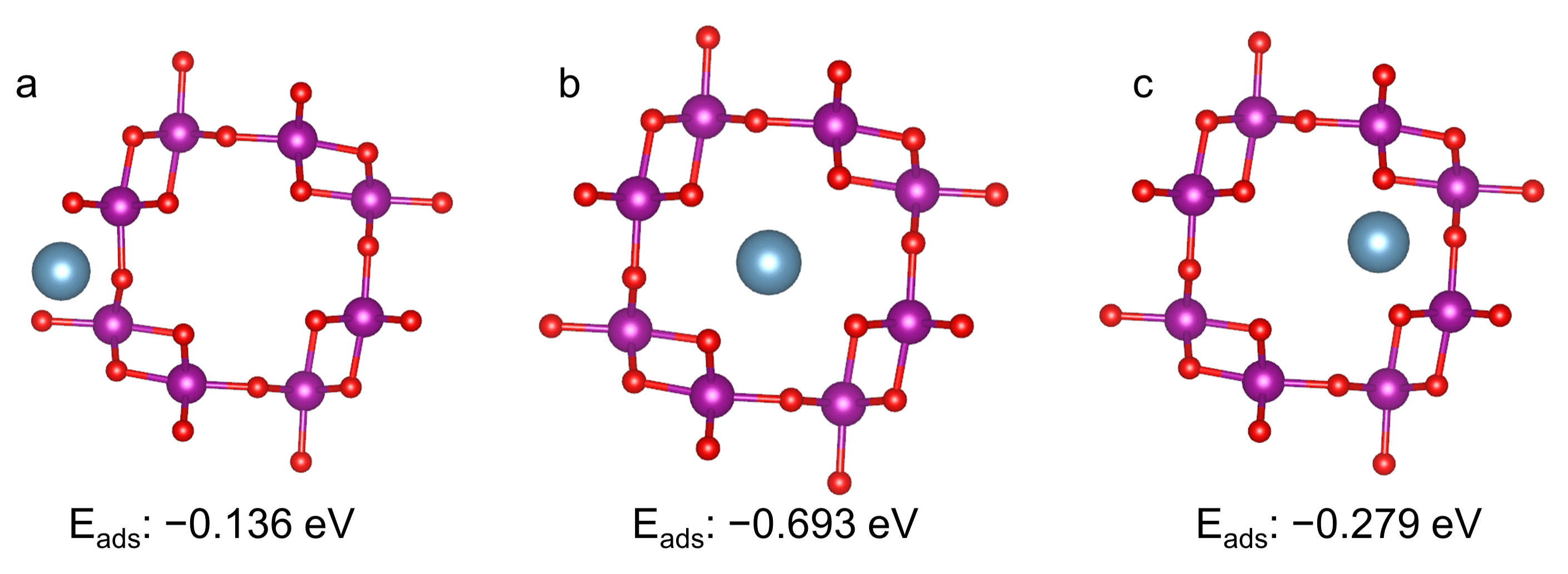


**Figure. S15** Optimized structure of calciated α-MnO_2_, with Ca^2+^ occupies (a) 4c site, (b) 2a site, and (c) 8h site of the 2×2 tunnels. The corresponding formation energies are presented as E_ads_.


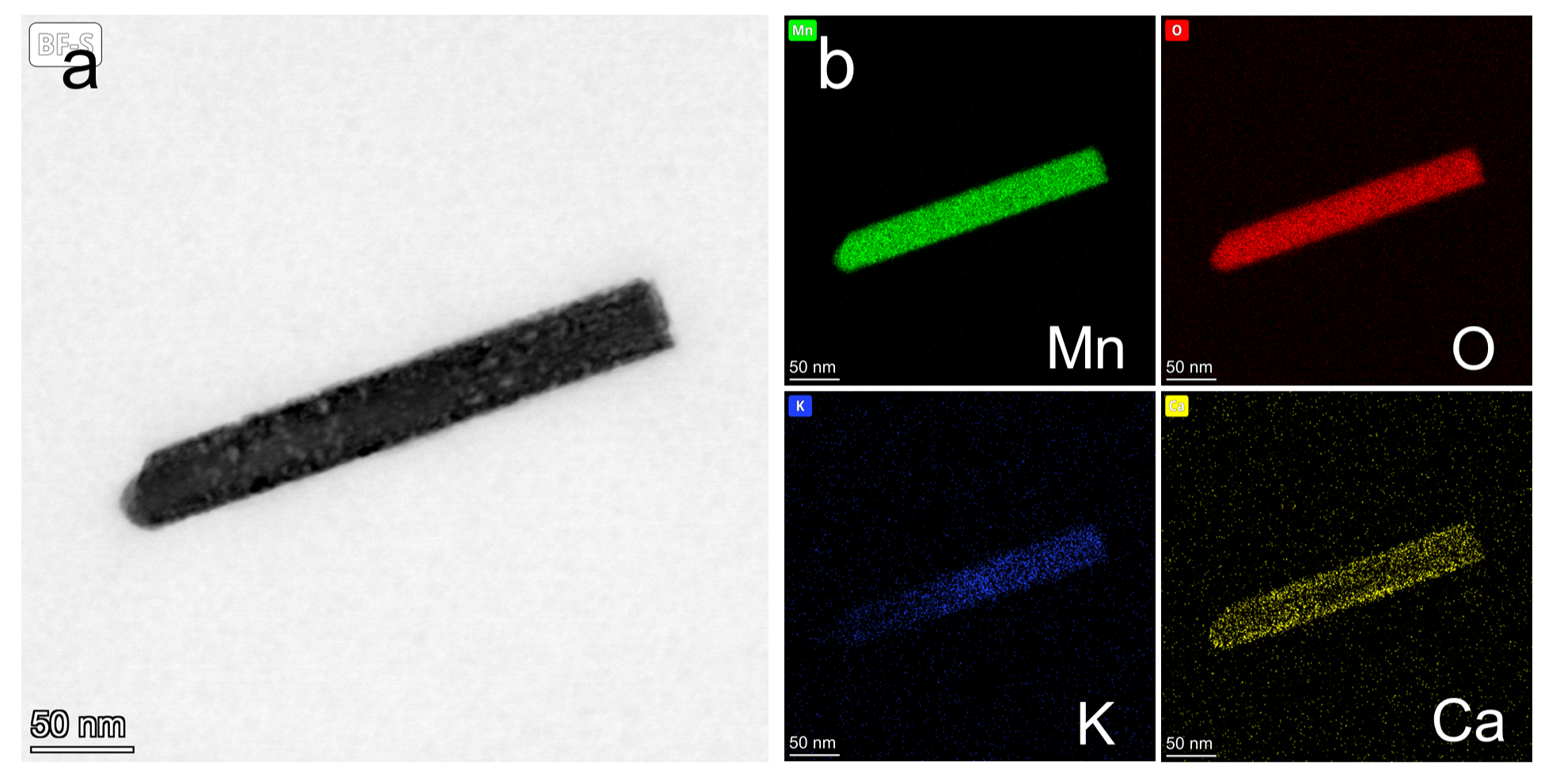


**Figure. S16** (a) TEM image, (b) elemental mapping images of the α-K_0.03_MnO_2_ cathode at the charged state with Ca anodes.





**Figure. S17** *Ex situ* XRD characterization of the α-K_0.03_MnO_2_ cathode during the tenth discharge/charge cycle in the Ca cells (at a current density of 10 mA g^−1^).


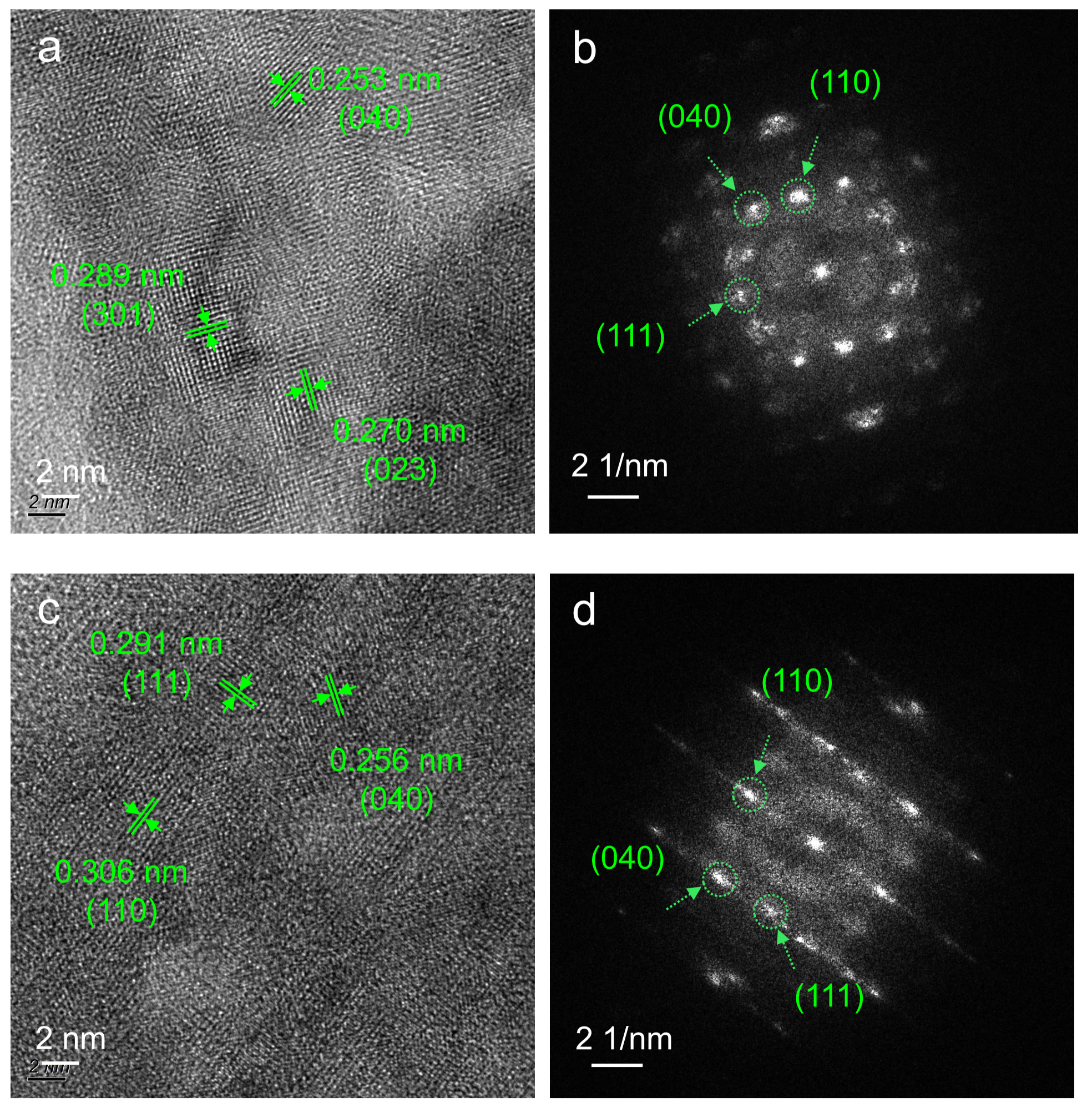


**Figure. S18** TEM characterization of the α-K_0.03_MnO_2_ cathode during the 10^th^ discharge/charge cycle in the Ca cells (at a current density of 10 mA g^−1^). (a) HRTEM image, and the corresponding (b) FFT patterns at discharge state; (c) HRTEM image, and the corresponding (d) FFT patterns at charge state.


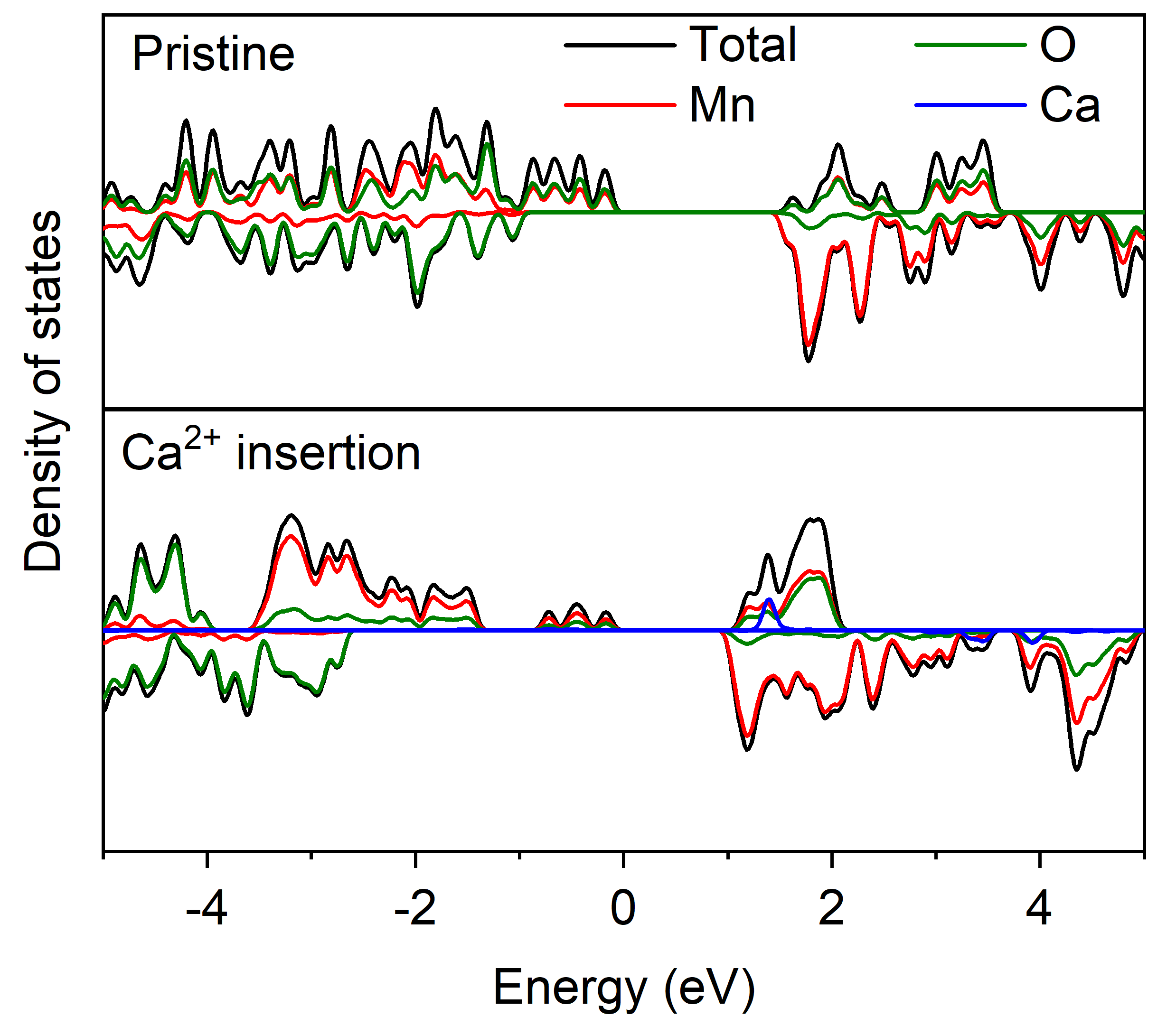


**Figure. S19** DOS of α-MnO_2_ and Ca^2+^ inserted α-MnO_2_.


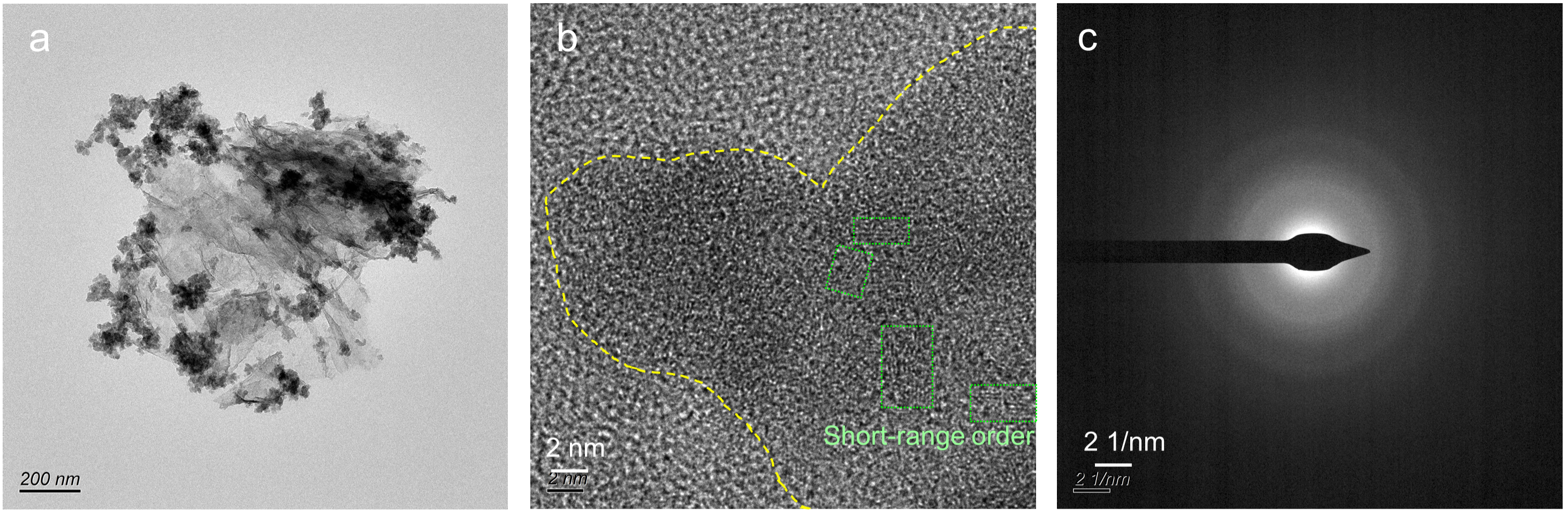


**Figure. S20** (a) TEM image of the u-MnO_2_@GO powders. (b) HRTEM image and (c) SAED pattern of the u-MnO_2_ nanoparticles.


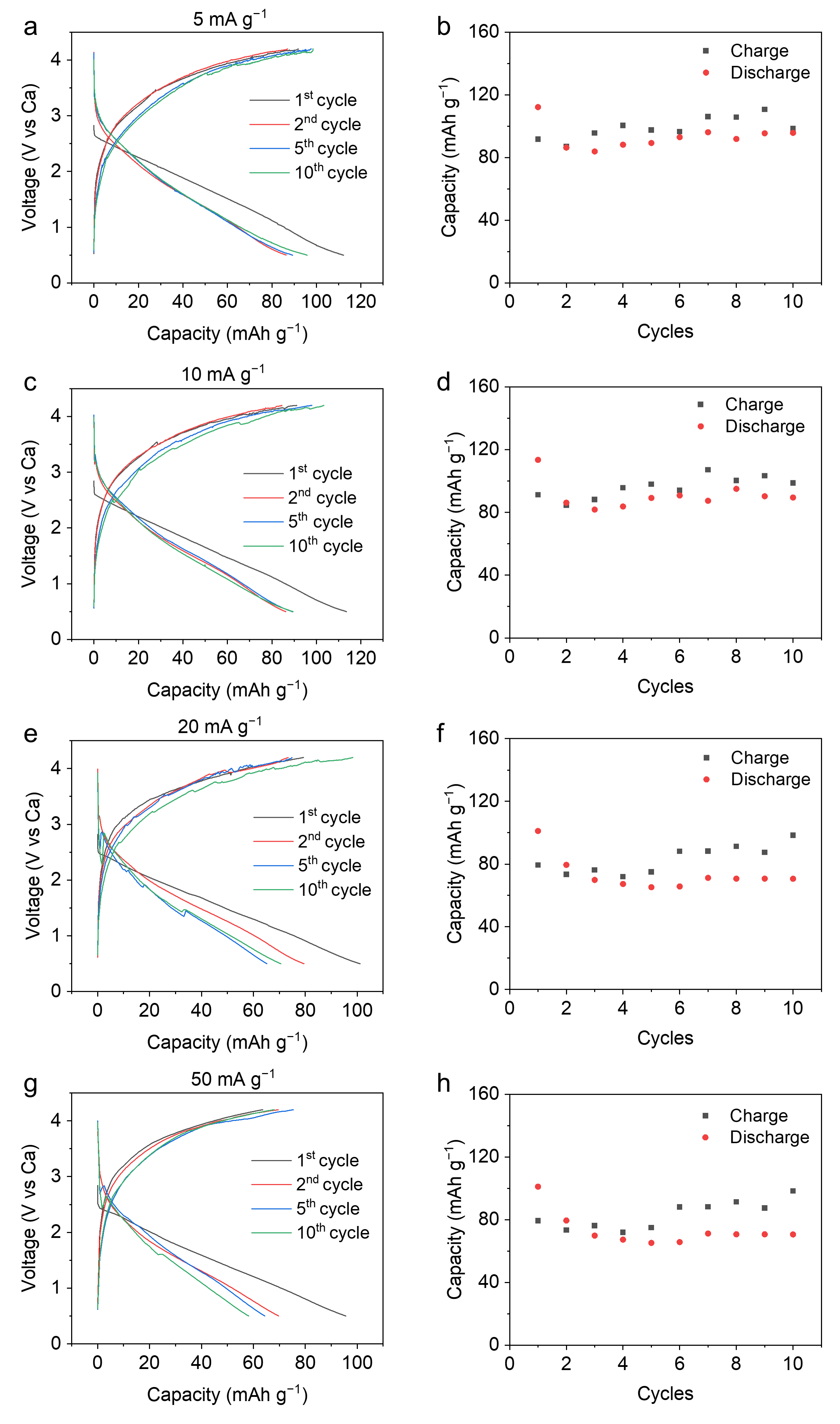


**Figure. S21** Cycling performance and GCD curve of the u-MnO_2_@GO cathodes cycled at (a, b) 5 mA g^−1^; (c, d) 10 mA g^−1^; (e, f) 20 mA g^−1^; (g, h) 50 mA g^−1^.


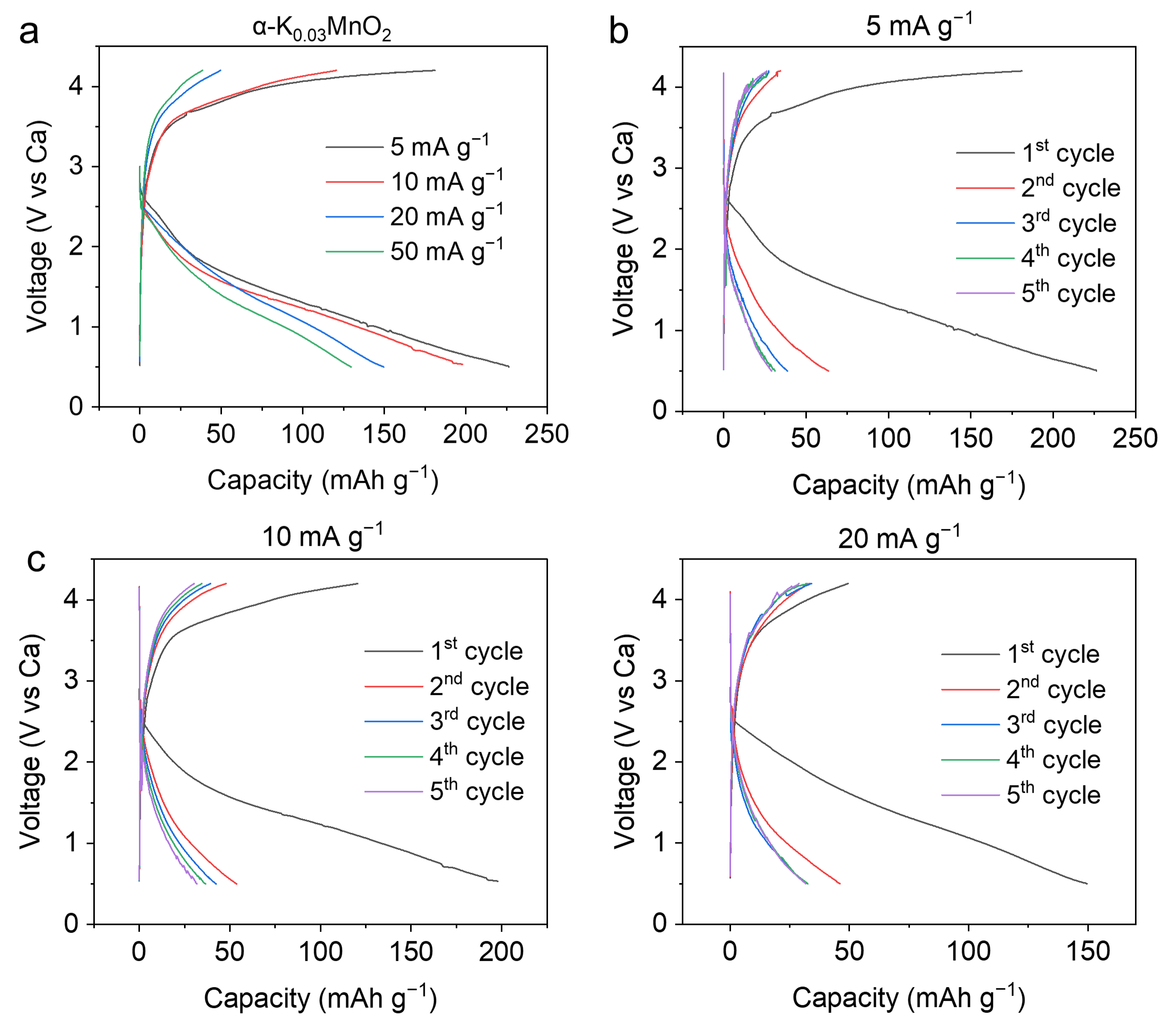


**Figure. S22** (a) Rate performance of α-K_0.03_MnO_2_ cathodes. GCD curves of α-K_0.03_MnO_2_ cathodes cycled at (b) 5 mA g^−1^, (c) 10 mA g^−1^, (d) 20 mA g^−1^.

The rate capability of α-MnO_2_ cathode was investigated by measuring the discharge-charge capacity on separate cathodes at progressively increasing current densities. It deviates from conventional methodology, where a single cell undergoes progressively increasing current densities. The reason for applying this method is that the irreversible phase transformation of α-MnO_2_ into CaMn_2_O_4_ during initial discharge-charge process causes drastic capacity decay in subsequent cycles regardless of current density (Figure 2b, Figure S22). Consequently, conventional rate testing cannot accurately assess Ca^2+^ diffusion kinetics within its tunnel structure.

**Figure. S23** CV curve of the u-MnO_2_@GO cathode at a scan rate of 0.2 mV s^−1^ in the cells with a Ca anode.

**Figure. S24** GCD curves of [α-K_0.03_MnO_2_@GO](mailto:α-K0.03MnO2@GO) cathode in Ca anode cells at 50 mA g^−1^.

**Figure. S25** XRD results of the u-MnO_2_@GO cathodes in the first discharge/charge cycle.

**Reference**

[1] S. Cui, D. Zhang, G. Zhang, Y. Gan, *J. Mater. Chem. A* **2022**, *10*, 25620-25632.

[2] Y. Yuan, C. Zhan, K. He, H. Chen, W. Yao, S. Sharifi-Asl, B. Song, Z. Yang, A. Nie, X. Luo, H. Wang, S. M. Wood, K. Amine, M. S. Islam, J. Lu, R. Shahbazian-Yassar, *Nat. Commun.* **2016**, *7*, 13374.

[3] Z. Li, O. Fuhr, M. Fichtner, Z. Zhao-Karger, *Energy Environ. Sci.* **2019**, *12*, 3496-3501.

[4] G. Kresse, J. Furthmuller, *Comput. Mater. Sci* **1996**, *6*, 15-50.

[5] G. Kresse, J. Furthmuller, *Phys Rev B* **1996**, *54*, 11169-11186.

[6] J. P. Perdew, K. Burke, M. Ernzerhof, *Phys. Rev. Lett.* **1996**, *77*, 3865-3868.

[7] G. Kresse, D. Joubert, *Phys Rev B* **1999**, *59*, 1758-1775.

[8] P. E. Blochl, *Phys Rev B* **1994**, *50*, 17953-17979.
